# A novel somatic mutation of SIN3A detected in breast cancer by whole-exome sequencing enhances cell proliferation through ERα expression

**DOI:** 10.1038/s41598-018-34290-1

**Published:** 2018-10-30

**Authors:** Kenji Watanabe, Shigeru Yamamoto, Syuiti Sakaguti, Keishiro Isayama, Masaaki Oka, Hiroaki Nagano, Yoichi Mizukami

**Affiliations:** 1 Institute of Gene Research, Yamaguchi University Science Research Center, Yamaguchi, 755–8505 Japan; 2 Department of Gastroenterological, Breast and Endocrine Surgery, Yamaguchi University Graduate School of Medicine, Yamaguchi, 755–8505 Japan; 3 Institute of Radioisotope Research and Education, Yamaguchi University Science Research Center, Yamaguchi, 755–8505 Japan; 4 Institute of Life Science, Yamaguchi University Science Research Center, Yamaguchi, 755–8505 Japan

## Abstract

Breast cancer is the most frequent tumor in women, and in nearly two-thirds of cases, the tumors express estrogen receptor α (ERα, encoded by *ESR1*). Here, we performed whole-exome sequencing of 16 breast cancer tissues classified according to *ESR1* expression and 12 samples of whole blood, and detected 310 somatic mutations in cancer tissues with high levels of *ESR1* expression. Of the somatic mutations validated by a different deep sequencer, a novel nonsense somatic mutation, c.2830 C>T; p.Gln944*, in transcriptional regulator switch-independent 3 family member A (SIN3A) was detected in breast cancer of a patient. Part of the mutant protein localized in the cytoplasm in contrast to the nuclear localization of ERα, and induced a significant increase in *ESR1* mRNA. The SIN3A mutation obviously enhanced MCF7 cell proliferation. In tissue sections from the breast cancer patient with the *SIN3A* c.2830 C>T mutation, cytoplasmic SIN3A localization was detected within the tumor regions where nuclear enlargement was observed. The reduction in *SIN3A* mRNA correlates with the recurrence of ER-positive breast cancers on Kaplan-Meier plots. These observations reveal that the SIN3A mutation has lost its transcriptional repression function due to its cytoplasmic localization, and that this repression may contribute to the progression of breast cancer.

## Introduction

Breast cancer is the most frequent primary tumor in women with an estimated 1.7 million cases diagnosed annually, and is the fifth leading cause of death among women. The female hormone estrogen (E2) is an important risk factor for the pathogenesis and progression of major breast cancers because increases in the lifetime exposure to E2 by life events such as early menarche, late natural menopause, and not bearing children are closely related to breast cancer^1^. E2 has two types of receptors, nuclear receptors and a membrane receptor. Estrogen receptor (ER) α is a nuclear hormone receptor with transcriptional activity that can lead to cell proliferation in response to its ligand. A membrane type of estrogen receptor, G protein-coupled estrogen receptor (GPER), isolated from a breast-cancer cell line, is induced by the hypoxic stimulation involved in the progression of various types of cancers^2–5^. The increased expression of GPER is related to aggressive breast cancers showing poor patient survival^6^.

Breast cancer is a heterogeneous disease that is classified into three subtypes: luminal, human epidermal growth factor receptor 2 (HER2)-enriched, and basal-like based on the presence of ERα (coded by the *ESR1* gene*)*, progesterone receptor (coded by the *PGR* gene) and HER2^1^. ERα-positive breast cancers belong largely to the luminal subtype that represents more than 60% of all breast cancers^7^. E2 blockers are used clinically as the first choice for endocrine therapy for luminal subtype breast cancers, and can reduce the risk of disease recurrence, but their use in the long term often results in the development of hormone-resistance through various mechanisms^8^. The elucidation of the molecular targets involved in ER expression in breast cancer is urgently needed to avoid the development of hormone-resistant breast cancers^8–10^.

Common features of tumors are their various genetic alterations, such as base substitutions, insertions/deletions, and rearrangements that are observed in relation to disease progression^11,12^. These mutations are divided into driver mutations that affect the disease and passenger mutations that have no effects on the disease^11^. Since the identification of driver mutations involved in tumor progression contributes to effective therapies and disease prognosis, mutations in breast cancers are being examined aggressively using deep DNA sequencers. In a recent report, likely driver mutations were newly identified in the exon regions of *AKT2*, *ARID1B*, *CASP8*, *CDKN1B*, *MAP3K1*, *MAP3K13*, *NCOR1*, *SMRCD1*, and *TBX3* based on analyses of the coding exon regions in 100 primary breast cancers in addition to the mutations previously identified in *AKT1*, *BRCA1*, *CDH1*, *GATA3*, *PIK3CA*, *PTEN*, *RB1*, and *TP53*^13^. Nik-Zainail *et al*. carried out a large-scale analysis of the whole genome of 560 breast cancers, and identified 916 probable driver mutations including *TP53*, *PIK3CA*, *MYC*, *CCND1*, *PTEN*, *ERBB2*, *ZNF703/FGFR1*, *RB1*, and *MAP3K1* as the most frequently mutated genes^14^. In luminal breast cancer subtypes defined by the presence of ERα, somatic mutations of *PIK3CA*, *MAP3K1*, *GATA3*, and *TP53* were found to be the most frequent mutations. To elucidate the pathogenetic factors activated by these genetic alterations, phosphoproteomic analysis by quantitative mass-spectrometry was carried out using 105 genetically annotated breast cancers^15^. CDC42BPG, an effector kinase for RHO family GTPase was clearly detected as an active kinase in the HER2-enriched subtype, and two kinases, PRKDC and SPEG were significantly observed in the basal-like breast cancer subtype. The driver mutations directly connected to the activation of *ESR1* expression remain unclear in luminal subtype breast cancers. The methylation of the promoter region in *ESR1* gene is observed through DNA methyl transferases (DNMTs) that insert a methyl group to cytosine of CpG residues in the ERα -negative breast cancers in contrast to the unmethylation in the transcriptional region of ERα -positive breast cancers. The methylation region is covered with methyl-CpG binding protein 2 (MeCP2), which associates with switch-independent 3 family member A (SIN3A) that can form the large complex with various transcriptional regulators including Histone deacetylases (HDACs). The large complex plays a key role in transcriptional silencing. In high-grade breast cancers, HDAC1 and DNA methyl transferase 3B are also recruited to the *ESR1* promoter by Twist expression, causing a reduction in the *ESR1* transcription level^16^. Recently, phosphoinositide-3-kinase (PI3K), coded by *PIK3CA* in which somatic mutations are frequently found, was found to phosphorylate histone methyltransferase KMT2D through Akt activation, which inhibits ERα-dependent transcription^17^. There is as yet no report elucidating the relationship between somatic mutations and increased *ESR1* expression in luminal subtype breast cancers, although epigenetic regulation appears to be involved in the expression of the *ESR1* mRNA^18^.

Here, we carried out whole-exome sequencing (WES) analysis of breast cancers classified by *ESR1* expression, and identified novel somatic mutations by exclusion of SNPs in the whole blood cells of patients with breast cancer. Of the somatic mutations validated by different types of DNA deep sequencers, genes directly related to *ESR1* expression were selected from an Ingenuity Pathway Analysis (IPA) pathway database. We found a novel nonsense mutation of a transcriptional regulator, SIN3A, that can associate with HDACs but has lost its binding region for MeCP2 in breast cancers showing high ERα expression. The somatic mutation *SIN3A* c.2830 C>T; p.Gln944* enhances the increase in *ESR1* expression, which, in turn, accelerates cell proliferation in MCF7 cells accompanied by cytoplasmic localization separate from the nuclear localization of ERα.

## Results

### Somatic mutations were detected in breast cancers with high levels of *ESR1* expression

**Figure 1:**
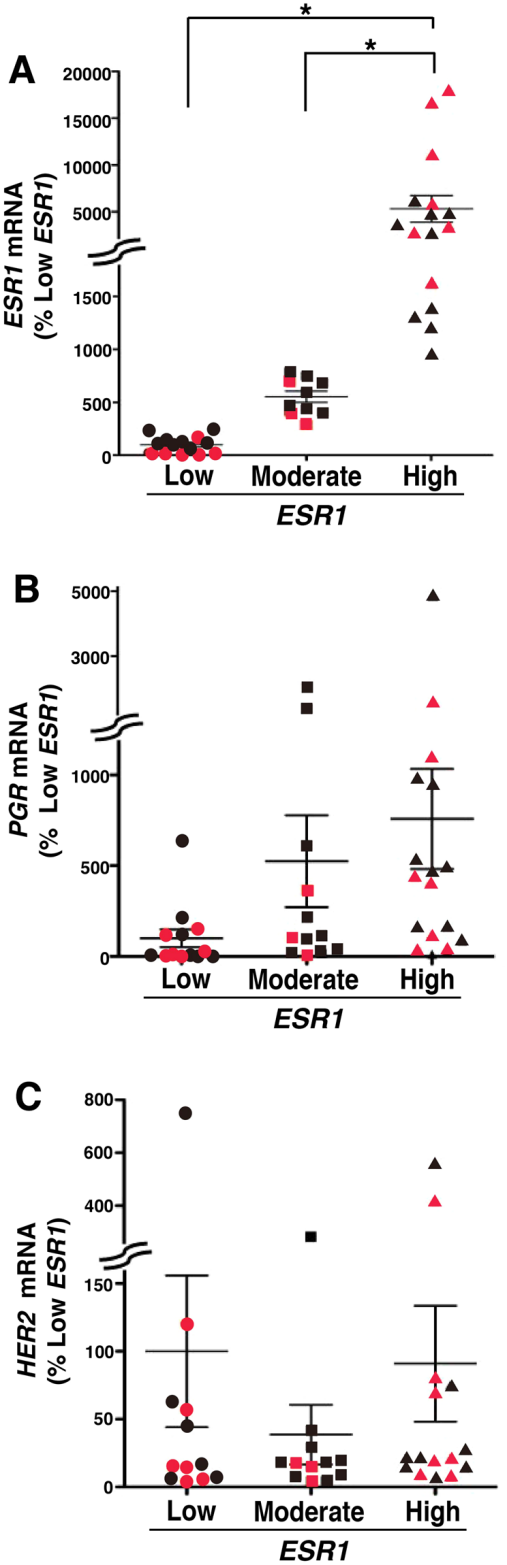
Expression of mRNAs for conventional biomarkers of breast cancers (**A-C**) Breast cancer tissues (n = 40) were minced and homogenized with a mixer mill, and total RNA was extracted and converted to cDNA by reverse transcriptase. The expressions of *ESR1* mRNA (**A**), *PGR* mRNA (**B**) and *HER2* mRNA (**C**) were determined by qPCR using the primers indicated in Supplemental Table 10S. The expression values are shown as comparative values for the mean of the low *ESR1* group. The tissues were classified according to the levels of *ESR1* mRNA expression into the high *ESR1* expression group greater than mean + 2 SD (high *ESR1*), the low *ESR1* expression group less than mean-2SD (low *ESR1*), or the moderate *ESR1* expression group (moderate *ESR1*) for all samples not characterized in the other groups. The expressions of mRNA were normalized to the expression of *GAPDH*. The red symbols show the samples used to whole exome sequences (WES). Statistical significance was determined by ANOVA with the Bonferoni *post hoc* test (*P < 0.05).

**Figure 2:**
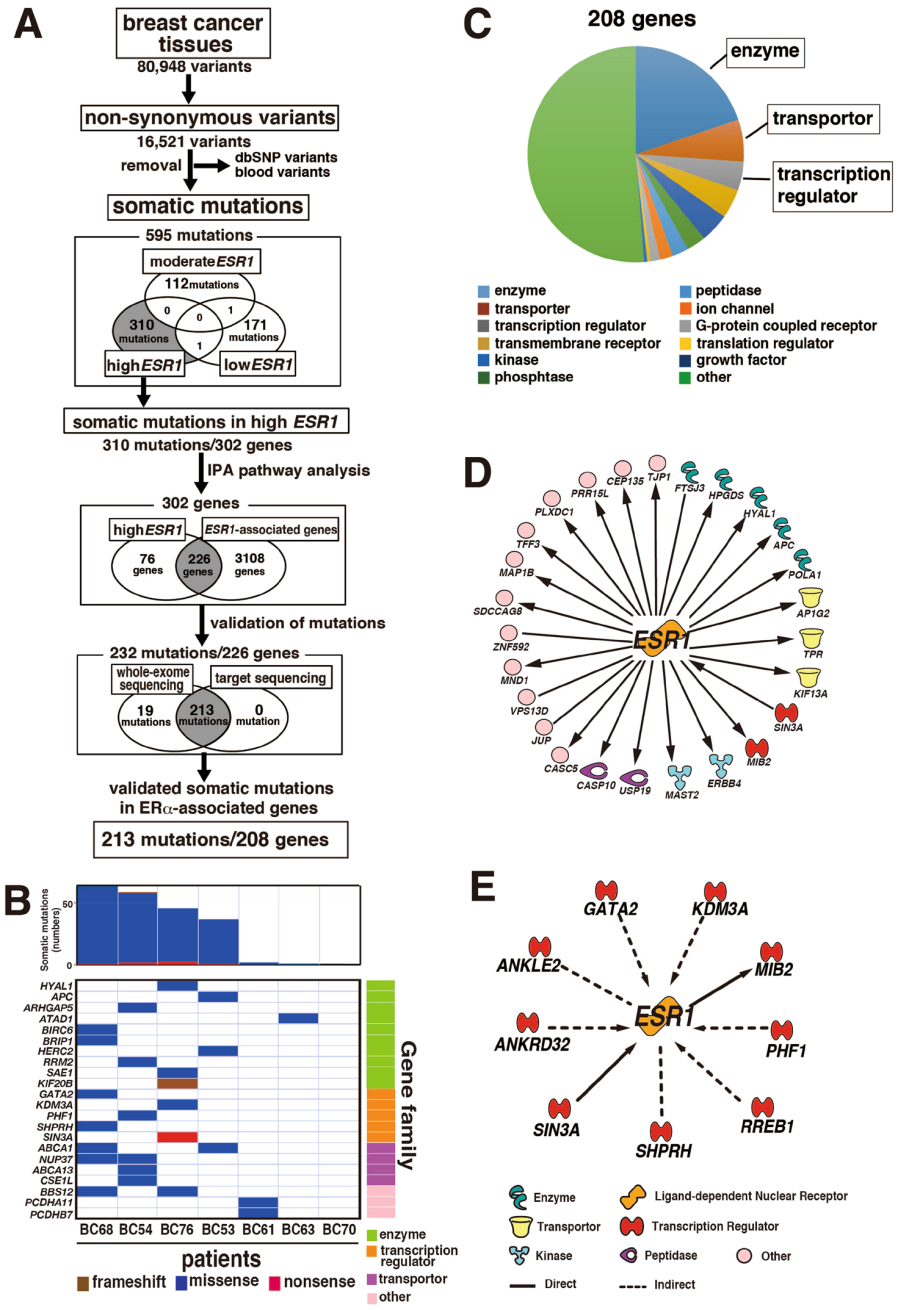
Detection of somatic mutations in breast cancers by WES analysis (**A**) The workflow for somatic mutation analysis in breast cancer tissues in the high *ESR1 *expression group is shown. WES analysis was performed using breast cancer tissue samples from the high *ESR1* group (n = 7), moderate *ESR1* group (n = 3), and low *ESR1* group (n = 6), resulting in 16,521 non-synonymous variants of 80,948 single nucleotide variants detected in all tissues. By removing SNPs detected in whole blood cells and SNPs on the dbSNP common database, 310 somatic mutations in the high *ESR1* group were identified in the tissues. Using the database on Ingenuity Pathway Analysis (IPA) software, 232 somatic mutations in 226 genes associated with *ESR1* were selected from the genes with somatic mutations. Finally, 213 somatic mutations in 208 genes were confirmed by target re-sequencing with an Ion PGM. (**B**) Genetic landscape of breast cancer tissues in the high *ESR1* expression group is shown. Numbers of somatic mutations in each tissue are shown in the upper bar graph with the indicated mutation types. The lower panel shows the mutation types in representative genes detected by WES analysis. The types of molecular functions are shown on the right side of the bottom panel. (**C**) The identified genes were classified according to their biological functions. (**D**) The direct network between *ESR1* and genes with somatic mutation is shown. **(E)** The direct and indirect network between *ESR1* and transcriptional regulators with somatic mutation is shown. The networks were revealed as pictures for genes and lines for biological relationship. Solid lines mean direct interaction, and dotted lines show indirect interactions between the genes. The shapes of genes indicate their molecular functions.

To examine somatic mutations involved in the high expression of *ESR1* in breast cancer tissues, tissues were classified into 3 groups according to their *ESR1* expression. Samples with *ESR1* levels higher than mean + 2 SD of all samples were classified into a high expression group (high *ESR1*, n = 15), and samples with *ESR1* levels lower than mean-2SD were classified into a low expression group (low *ESR1*, n = 14). Samples not classified into the high *ESR1* or low *ESR1* groups were assigned into the moderate expression group (moderate *ESR1*, n = 11). The expression levels of *ESR1* mRNA in the high *ESR1* group were more increased than 50-fold higher than those in the low *ESR1* group (Fig. 1A). The expression of *PGR* mRNA in the high *ESR1* group appeared to be higher than that in the low *ESR1* group (Fig. 1B), but there were no significant differences among the groups (Fig. 1B). *HER2* mRNA expression in the samples was unaffected by the level of *ESR1* expression (Fig. 1C). WES analysis of genomic DNA extracted from tissues of breast cancers classified by *ESR1* was carried out with a deep sequencer SOLiD5500. To remove variants unrelated to the disease, WES analysis of blood collected from patients with the corresponding breast cancers in each group were also analyzed as negative controls except for 4 uncollected samples. The measured reads were aligned on human reference sequences (hg19) using CLC Genomics Workbench software; the numbers of bases mapped on the exon regions in each group were approximately 2.1 Gb/sample. The average depth of sequencing in the exon region for high *ESR1*, moderate *ESR1*, and low *ESR1* samples was 45, 50 and 51, respectively (Supplementary Table 1S). In the mapped sequences, 742,034 variants including 75,783 non-synonymous variants were detected in all breast cancer samples. Variants also detected in blood samples or listed in dbSNP (common, ver.150) were removed from the non-synonymous variants, leaving a total of 39,734 variants detected as somatic mutations related to breast cancer. Of these somatic mutations, the high *ESR1* group samples had 19,228 mutations while 18,485 mutations were included in the low *ESR1* and moderate *ESR1* group samples, and 754 mutations were detected as common mutations in three groups (data not shown). The mapping parameters were modified to detect only point mutations as described under Materials & Methods. Under the analytical conditions used, 16,521 non-synonymous variants were detected in all samples of breast cancer including 80,948 variants. After removal of the variants detected in blood samples and listed in dbSNP (common, ver.150), 595 mutations were detected in the samples as novel somatic mutations. Of these somatic point mutations, 310 mutations were in the high *ESR1* group and 171 mutations were in the low *ESR1* group (Fig. 2A).

### Somatic mutations related to ERα were selected using the IPA database

To select the somatic mutations evoking the up-regulation of *ESR1* expression, we examined whether the genes containing somatic mutations are included as genes associated with ERα in the IPA database of known protein interactions. Of 302 genes with somatic mutations detected in breast cancers, 226 genes were coincident with genes that interact with ERα in the IPA database pathway (Fig. 2A).

### Somatic mutations were validated by target sequencing with another deep sequencer

To confirm the somatic mutations detected by WES, all of the detected genes were amplified by a custom panel consisting of 359 primer sets, and the amplicons were analyzed with Ion Personal Genome Machine (PGM). The average depth of the sequencing was more than 1200-fold for all of the targeted genes, and 213 mutations in 208 genes were confirmed as point mutations identical to those in WES (Fig. 2A, Supplementary Table 2S and 3S). Most of the 19 mutations that were not detected by target re-sequencing may have been false-positives, since coverage of most of the detected sequences was less than 6 reads, and their levels were near the detection limit of the experiment. Among the 208 confirmed genes, mutations in *NUP37*, *C18orf8*, and *BBS12* were detected in 2 cancer tissues. The genes containing somatic mutations belonged mainly to 3 categories, enzymes, transporters, and transcription regulators (Fig. 2B,C, Supplementary Table 4S and 5S). The following genes were included among transcription regulators, *SIN3A*, *GATA2*, *KDM3A*, *MIB2*, *ANKLE2*, *ANKRD32*, *PHF1*, *RREB1* and *SHPRH* (Fig. 2D,E). Of the transcriptional regulators, *GATA2*, *PHF1* and *SIN3A* were previously reported to be involved in breast cancer (Fig. 2D,E). We focused on the relationship between the nonsense point mutation c.2830 C>T; p.Gln944* in *SIN3A* and *ESR1* expression in breast cancer cells, because SIN3A, which is coded by the gene for *SIN3A*, works as a transcriptional repressor of ERα as reported^19,20^.

### *ESR1* mRNA is up-regulated by the *SIN3A* c.2830 C>T; p.Gln944* mutant

**Figure 3:**
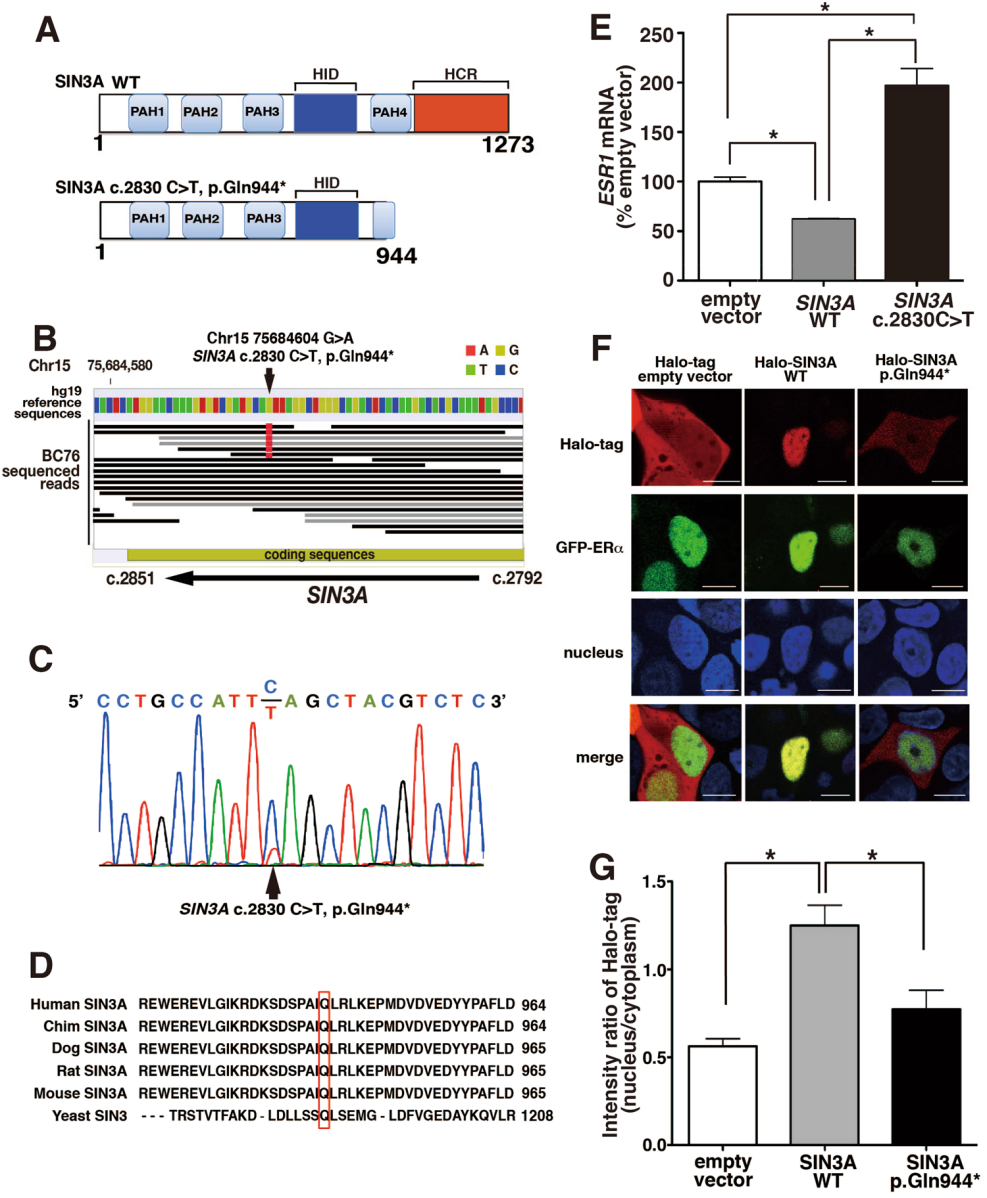
Molecular structure and biological functions of *SIN3A* c.2830 C>T; p.Gln944* (**A**) Molecular structures of *SIN3A*-WT and the *SIN3A* c.2830 C>T; p.Gln944* nonsense mutant are shown in the upper panel and lower panel, respectively. (**B**) The sequenced reads of the coding region in *SIN3A* using SOLiD5500 were shown. (**C**) The point mutation of *SIN3A* c.2830 C>T; p.Gln944* as detected in breast cancer tissue was confirmed by Sanger sequencing. (**D**) Alignments of amino acid sequences adjacent to Gln944 in SIN3A in various species. The PAH4 domain on SIN3A is highly conserved in various species from yeasts to humans. (**E**) *ESR1* mRNA expression was increased in MCF7 cells transfected with the *SIN3A* c.2830 C>T; p.Gln944*. MCF7 cells were transfected with the indicated vectors together with pMACS4.1 and were incubated with 100 nM E2 for 24 hrs. The *ESR1* mRNA was measured by qPCR after the separation with an autoMACS pro. The data represent the means ± SE obtained in 4 independent experiments. Statistical significance was calculated by ANOVA with the Tukey–Kramer multiple comparison test (*P < 0.05). (**F**) The intracellular localizations of SIN3A were observed in MCF7 cells transfected with the *SIN3A* mutant. MCF7 cells were transfected with the indicated vectors together with GFP-tagged ERα expression vector. The cells were incubated in culture medium containing TMR ligand for Halo-tag stain and Hoechst33342 for nucleus stain at 24 hrs after transfection. SIN3A (red), ERα (green) and nucleus (blue) were observed under a confocal microscope LSM710. The pictures show representative results obtained from 6 independent experiments. Scale bar shows 20 µm. (**G**) Changes in intracellular localizations of SIN3A-WT or SIN3A c.2830 C>T; p.Gln944* were calculated from the total fluorescence intensities in the nucleus and the cytoplasm using an IN Cell analyzer 2000 and IN Carta software. The data represent the means ± SE obtained from 190 cells transfected with empty vector, 183 cells transfected with *SIN3A*-WT, and 341 cells transfected with *SIN3A* c.2830 C>T; p.Gln944*. Statistical significances were determined by ANOVA with the Tukey–Kramer multiple comparison test (*P < 0.05).

SIN3A is a protein with a molecular mass of 145 kDa comprosing 1273 amino acids, and containing four paired amphipathic α-helix (PAH) motifs, an HDAC interaction domain (HID) and a highly conserved region (HCR) (Fig. 3A). In the deep sequencers, approximately 30% of the *SIN3A* sequence reads in the tissue samples showed a point mutation that changed a cytosine nucleotide into a thymine nucleotide at position 2830 (c.2830 C>T). This mutation leads to a change from a glutamine into a stop codon at position 944 (p.Gln944*), and may be heterozygous, since the largest frequency of alleles in the tumor was within the range 50% to 60% (data not shown). As shown in Fig. 3B,C, the *SIN3A* c.2830 C>T mutation was re-confirmed by Sanger sequencing, showing the same fraction as the deep sequencers. Among 40 breast cancer samples examined by Sanger sequencing, one sample contained a nonsense mutation, c.2830 C>T in *SIN3A*. In the cohort database International Cancer Genome Consortium (ICGC, https://icgc.org), somatic mutations of SIN3A were found in 12.1% of ER-positive breast cancers (n = 569), 4.3% of triple negative (n = 141), or 9.7% of Her2-positive breast cancers (n = 72) (Supplementary Table 6S). Nonsense and frameshift mutations that deleted the C-terminal region of SIN3A were detected in 3 cases (0.52%) (Supplementary Table 7S). No nonsense or frameshift mutations resulting in the deletion of the C-terminus of SIN3A were detected in triple negative (n = 141, Supplementary Table 8S) or Her2-positive breast cancers (n = 72, Supplementary Table 9S). The mutation is present within the PAH4 domain, which is a conserved region in organisms from yeast to mammals (Fig. 3D), and leads to the deletion of the downstream sequence from the point mutation including the latter part of the PAH4 domain and the HCR region (Fig. 3A)^21^. This nonsense mutation of SIN3A may affect *ESR1* expression because the deleted regions bind to various regulators to act as a transcriptional repressor. We observed the expression of the *ESR1* mRNA in a breast cancer cell line, MCF7 cells, transfected with the *SIN3A* mutant in the presence of 100 nM E2. The *SIN3A*-wild type (WT)-transfected MCF7 cells showed a significantly decreased expression of *ESR1* mRNA as compared with cells transfected with empty vector at 24 hrs after transfection (Fig. 3E). In *SIN3A* c.2830 C>T; p.Gln944* expressing cells, *ESR1* expression was significantly increased as compared with those in cells transfected with *SIN3A*-WT and empty vector. The background SIN3A protein may have little influence on the functions of the exogenous SIN3A mutant because the amount of endogenous SIN3A protein was less than 1% as compared with the amount of exogenous SIN3A mutant protein (data not shown). These findings indicate that the function of SIN3A as a transcriptional repressor of *ESR1* expression is interfered with by the SIN3A c.2830 C>T; p.Gln944* mutant.

### SIN3A p.Gln944* mutant localizes in the cytoplasm of MCF7 cells

It was previously reported that SIN3A co-localizes with ERα in the nucleus, and acts as a transcriptional repressor in MCF7 cells^19^. To elucidate the mechanism of the up-regulation of *ESR1* mRNA by SIN3A p.Gln944* mutant, we observed the intracellular localization of the SIN3A mutant in MCF7 cells. Halo-tagged SIN3A-WT was present in the nucleus as indicated by a Hoechst33342 a nuclear marker, and the staining was identical to that of GFP-tagged ERning four paired a (Fig. 3F). In contrast to the localization of SIN3A-WT, the SIN3A mutant was observed not only in the nucleus, but also in the cytoplasm, although GFP-ERning four paired a remained confined to the nucleus (Fig. 3F). This result was supported by high-throughput analysis of the data determined from the pictures (Fig. 3G and Supplementary Fig. 1S). The findings indicate that the presence of the SIN3A mutant in the cytoplasm may lead to the reduction in its activity as a transcriptional repressor of *ESR1* expression.

### SIN3A p.Gln944* mutant increases cell proliferation through *ESR1* expression

**Figure 4:**
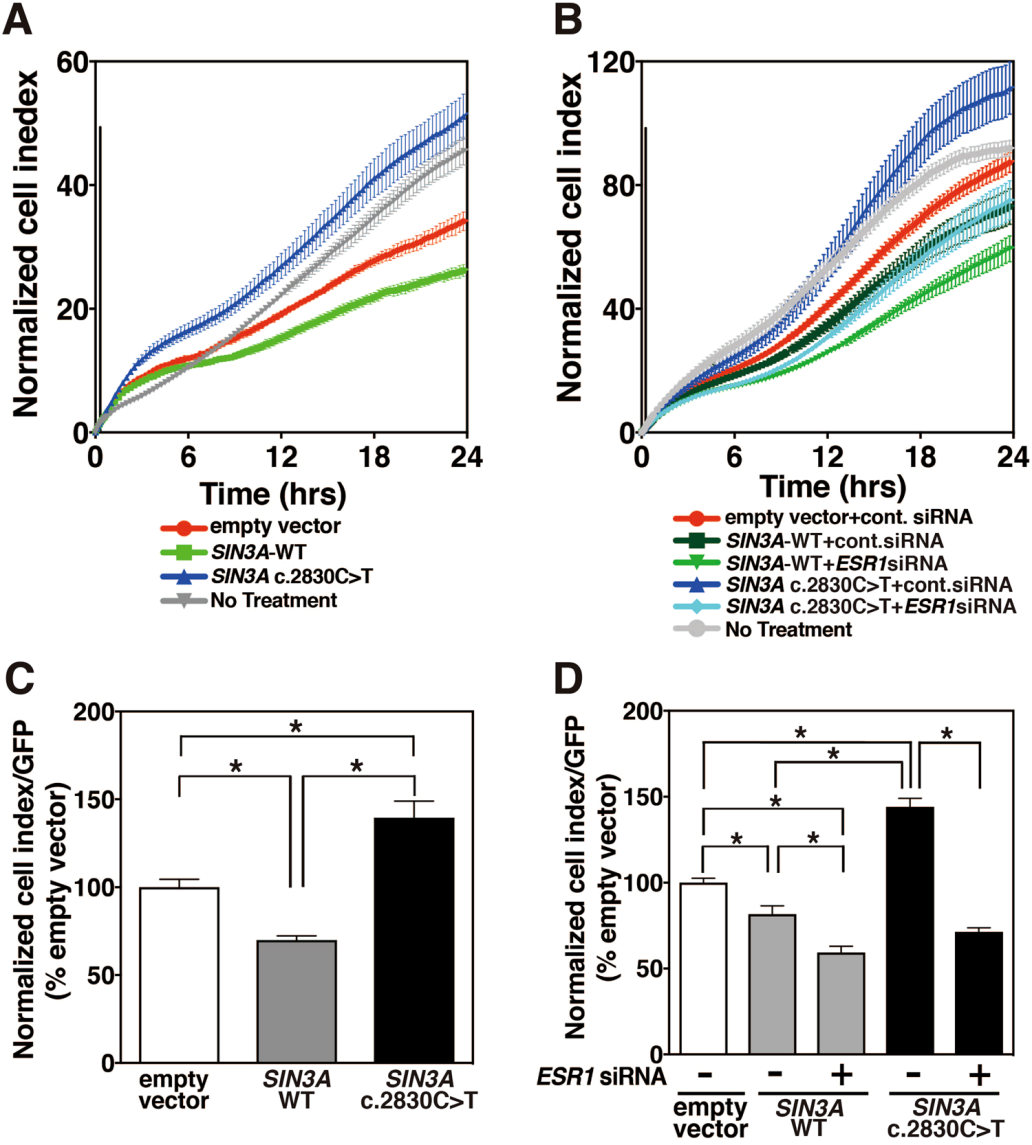
Impedances of MCF7 cells transfected with *SIN3A* c.2830 C>T (**A**) MCF7 cells were transfected with indicated vectors together with pMACS 4.1 vector that expresses a truncated *CD4* gene and pGFP expression vector for normalization for gene expressing cells. The untransfected MCF7 cells were also used as control cells. After transfection, the cells were cultured for 24 hrs and the transfected cells were separated with anti-CD4 antibody magnet beads. The separated cells were seeded at a density of 2.5 × 10^5^ cells/ml on a 16 well E-plate, and were incubated in the presence of 100 nM E2. Cell impedances were then continuously monitored at 15 min intervals for 24 hrs using the xCELLigence system. The data represent the means ± SE determined from 3 wells, and the figure shows representative data obtained from 2 independent experiments. (**B**) MCF7 cells were transfected with indicated vectors together with pMACS 4.1 vector that expresses a truncated *CD4* gene and pGFP expression vector for normalization for gene expressing cells in the presence of control siRNA or siRNA for *ESR1*. The untransfected MCF7 cells were also used as control cells. After transfection, the cells were cultured for 24 hrs and the vector-transfected cells were separated with anti-CD4 antibody magnet beads. The separated cells were seeded at a density of 2.5 × 10^5^ cells/ml on a 16 well E-plate, and were incubated in the presence of 100 nM E2. Cell impedances were then continuously monitored at 15 min intervals for 24 hrs using the xCELLigence system. The data represent the means ± SE determined from 6 wells, and the figure shows representative data obtained from 3 independent experiments. (**C**) The normalized cell indexes were determined from data of Fig. 5A, and GFP fluorescence intensities were calculated using an IN Cell analyzer 2000 at 24 hrs from the start. The data represent the means ± SE determined from 3 wells. Statistical significances were determined by ANOVA with the Tukey–Kramer multiple comparison test (*P < 0.05). (**D**) The normalized cell indexes were determined from data of Fig. 5B as similar to Fig. 5C.

To examine whether the up-regulation of *ESR1* expression induced by SIN3A p.Gln944* is involved in cell proliferation, we observed changes in cell area that represent cell proliferation by measuring cell impedance using an electrode in real time in the presence of 100 nM E2. The cell impedances of SIN3A mutant-expressing MCF7 cells were obviously increased over the levels of cells transfected with the empty vector and *SIN3A*-WT (Fig. 4A). The transfection of the siRNA for *ESR1* into MCF7 cells resulted in a significant reduction in *ESR1* mRNA expression (Supplementary Fig. 2S). The cell impedance that was increased by the SIN3A mutant was reduced to the level in cells containing SIN3A-WT introduced with control siRNA by the introduction of the siRNA for *ESR1* (Fig. 4B). To remove the effects of variations in exogenous SIN3A expression, the levels of cell impedance at 24 hrs were normalized by the fluorescence contents observed under the fluorescence microscope IN Cell analyzer 2000 using a GFP expression vector. The data in SIN3A-WT and SIN3A mutant were mostly consistent with those of the time-lapse impedances (Fig. 4C,D). These observations indicate that SIN3A p.Gln944* enhances cell proliferation through increased *ESR1* expression.

### SIN3A c.2830 C>T; p.Gln944* was observed in the cytoplasm of breast cancer tissue removed from a patient

**Figure 5:**
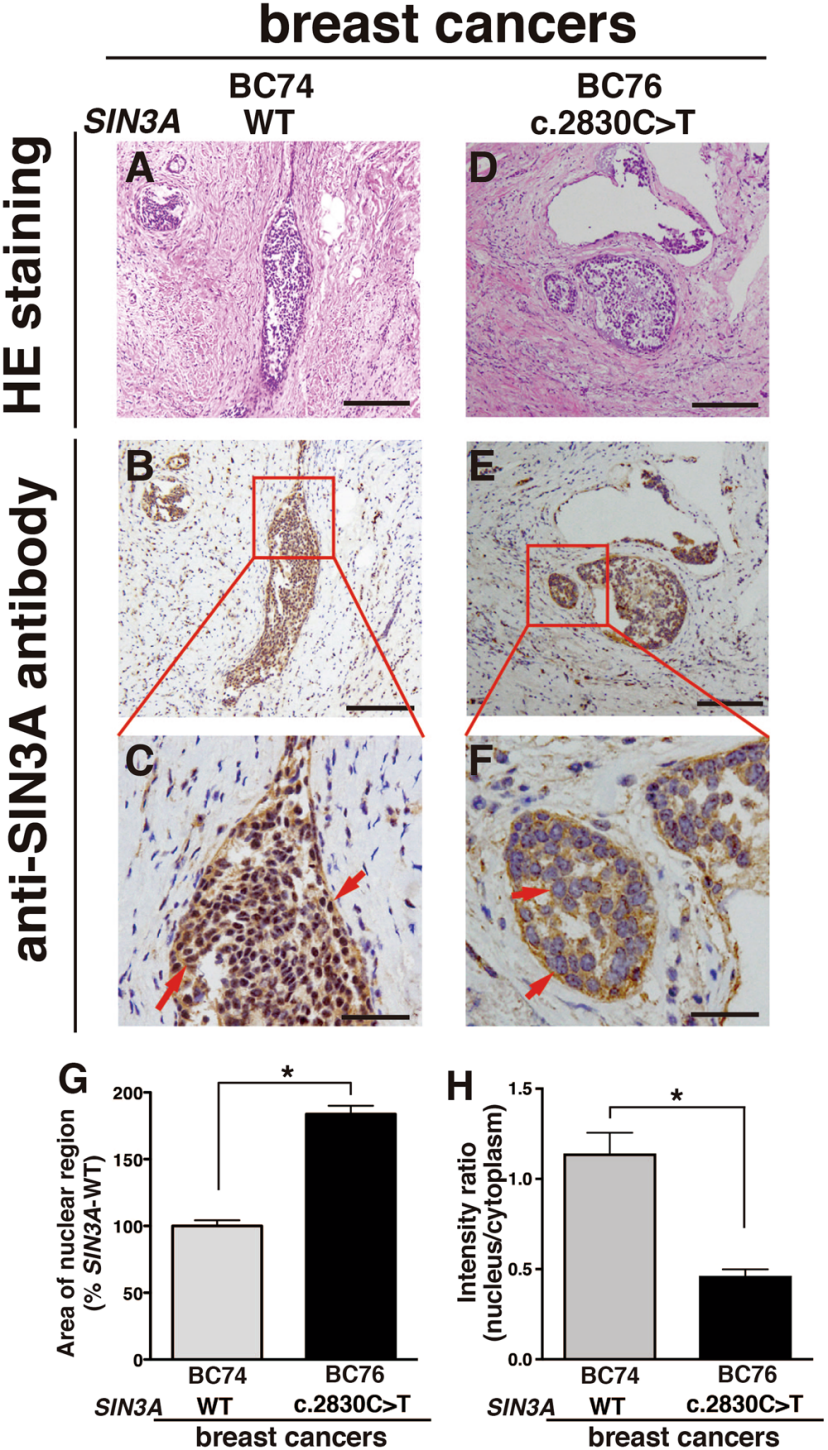
Intracellular distribution of SIN3A p.Gln944* in breast cancer tissues Tissues with *SIN3A*-WT (**A**–**C**) or *SIN3A* c.2830 C>T (**D**–**F**) were fixed with 4% paraformaldehyde, and cut into 4 µm thick sections after embedding in paraffin blocks. The sections were stained with hematoxylin and eosin (**A**–**D**), and then stained with anti-SIN3A antibody for the N-terminal region concurrently with counterstaining with hematoxylin to detect the nuclei (**B**–**F**). The boxed regions in the pictures stained with anti-SIN3A antibody (**B**–**E**) are magnified 4-fold (**C**–**F**). The arrows indicate the typical nuclear regions. The scale bars are 200 µm (**A**–**E**) and 50 µm (**C**–**F**). For the quantitative analysis of the areas of the nuclear regions and SIN3A localization on the tissues slides, breast cancer tissues containing *SIN3A*-WT (BC74) or *SIN3A* c.2830 C>T (BC76) were fixed with 4% paraformaldehyde, embedded in paraffin, and sliced into 4 µm thick sections. The sections were independently stained with hematoxylin to detect nuclei, eosin to detect the cells, or anti-SIN3A antibody. The areas of the nuclear regions in 50 cells (**G**) and the total intensities of SIN3A in each region in 20 cells (**H**) were determined using Metamorph software. The data represent the means ± SE. Statistical significances were determined by two-tailed Student’s t test (*P < 0.05).

To examine the distribution of SIN3A in tissues removed from patients with breast cancers, immunohistochemical observation was carried out using the antibody against the N-terminal peptide of SIN3A as an antigen to detect the WT and the mutant deleting C-terminal regions. SIN3A staining in tissue sections of the SIN3A-WT coincided with cancer lesions observed in clusters similar to the staining of the mutant SIN3A (Fig. 5A,B), but SIN3A staining was strongly detected within the nuclei under magnified imaging, and the nuclear staining of hematoxylin disappeared due to the staining overlapped with SIN3A staining (Fig. 5C). In the breast cancer tissue containing the c.2830 C>T mutation of *SIN3A*, SIN3A staining was strongly observed within the regions of the cancer lesions (Fig. 5D,E), and the magnified imaging showed the distribution of SIN3A mutant to be cytoplasmic, since the blue staining by hematoxylin used as a counterstain was clearly detected in the nuclear regions (Fig. 5F). The progressive cancer lesions with enlarged nuclei were observed in clusters covering approximately 30% of the region within the sections. The areas of the nuclear regions on the slide containing the SIN3A mutant were increased nearly 2-fold as compared with those containing SIN3A-WT (Fig. 5G). Quantitative analysis of SIN3A staining in each region of the slides also showed SIN3A staining on the slide for the SIN3A mutant in both the nucleus and cytoplasm in contrast to staining confined to the nucleus on the slide for SIN3A-WT (Fig. 5H). In sporadic breast cancers, similar data were observed (Supplementary Fig. 3S). These observations indicate that the progression of cancer cells through the expression of ERα was enhanced due to the loss of transcriptional suppression caused by the cytoplasmic localization of the SIN3A p.Gln944* mutant.

### The reduction in *SIN3A* mRNA expression affects the relapse-free survival curves of patients with ERα-positive breast cancer

**Figure 6:**
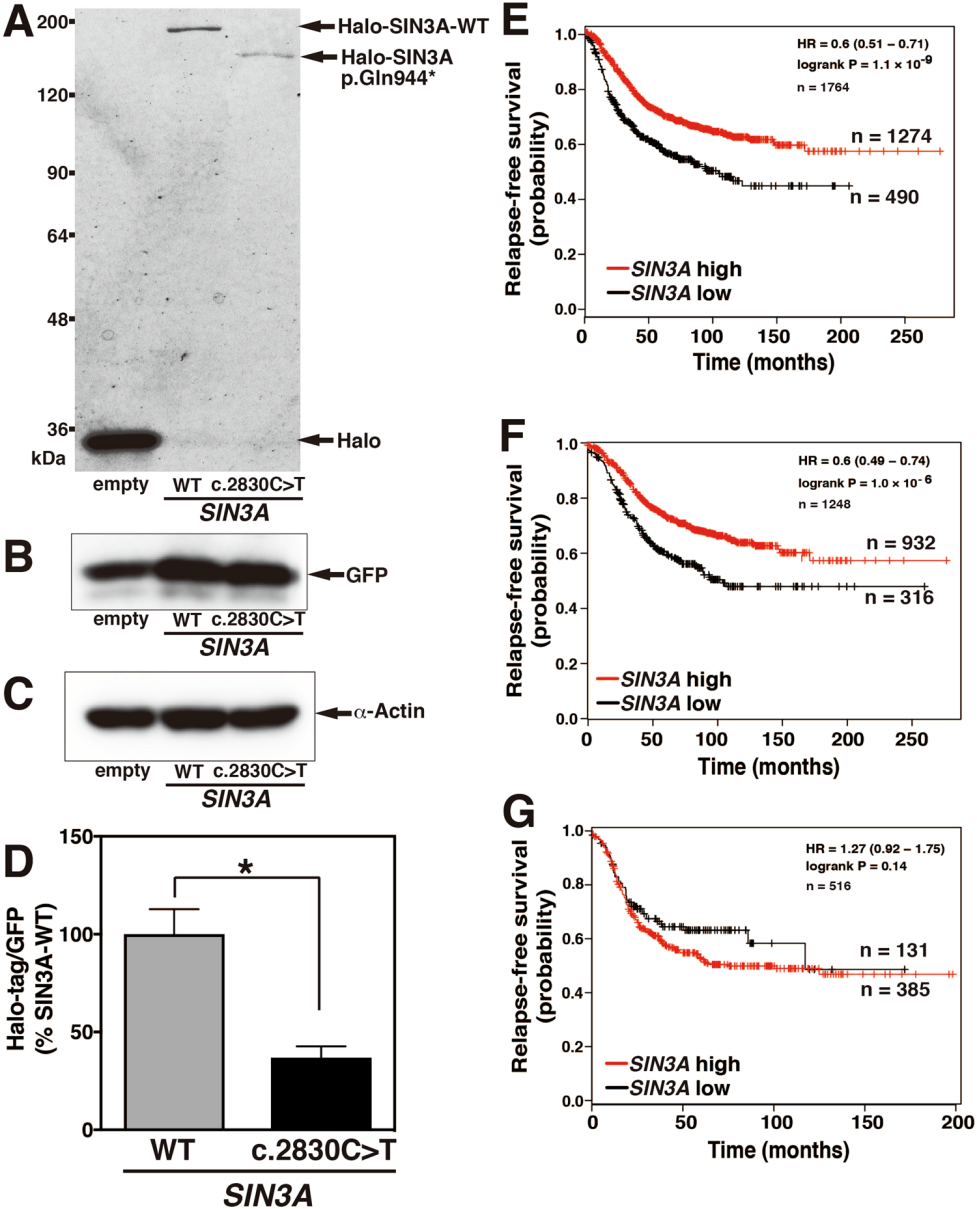
Expression of SIN3A p.Gln944* protein in MCF7 cells and relapse-free survival curves of patients with breast cancer according to *SIN3A* mRNA expression (**A–D**) MCF7 cells were transfected with *SIN3A*-WT, *SIN3A* c.2830 C>T, or empty vector together with pMACS4.1 and pGFP, and the cells were cultured for 24 hrs. The vector-transfected cells were separated with anti-CD4 antibody magnet beads. The cells were extracted, and were added Halo-tag ligand TMR (**A**). The samples were subjected to SDS-PAGE or immunoblotting using anti-GFP antibody (**B**) or anti-α-Actin antibody (**C**). The fluorescence of Halo-tag ligand on SDS-PAGE was detected with a Typhoon FLA9000. The bands corresponded to the indicated proteins were analyzed by Image Quant QL. The figure shows representative data obtained from 3 independent experiments. The data represent the means ± SE. Statistical significances were determined by two-tailed Student’s t test (*P < 0.05) (**D**). (**E**–**G**) Relapse-free survival curves were drawn for the high *SIN3A* expression group (red) and low *SIN3A* expression group (black) for all (**E**, n = 1764), ER-positive (**F**, n = 1248), or ER-negative (**G**, n = 516) breast cancer patients using the Kaplan-Meier Plotter.

To confirm the molecular mass of exogenous SIN3A protein, the proteins in the MCF7 cell transfected with Halo-SIN3A-WT or Halo-SIN3A c.2830 C>T mutant were detected by SDS-PAGE using fluorescence-conjugated Halo-tag ligand. The molecular mass of SIN3A-WT was observed around the approximately 185 kDa, which was corresponded to the calculated molecular mass (Fig. 6A). The band of the C-terminal deleted SIN3A p.Gln944* mutant was detected around 150 kDa consistent with the theoretical molecular mass. The band density of SIN3A p.Gln944* was obviously decreased that of SIN3A-WT (Fig. 6A,D, and Supplementary Fig. 4S), although the bands of GFP used as the expression control and α-Actin used as loading control were almost constant among the samples (Fig. 6B and C). The level of *SIN3A* mRNA expression in the breast cancer tissue with *SIN3A* c.2830 C>T appeared to be lower than in those with *SIN3A*-WT (Supplementary Fig. 5S), and *SIN3A* mRNA in the *SIN3A* mutant samples included approximately 20% *SIN3A* mutant sequence (data not shown). The cytoplasmic localization of the SIN3A mutant causes its loss of function resulting in cell proliferation, and the expression level of *SIN3A* mRNA may influence the survival curves for patients with breast cancers. The overall and relapse-free survival curves relative to *SIN3A* expression were drawn for patients classified according to *ESR1* expression on the Kaplan-Meier Plotters website. The overall survival curve for all patients revealed a lower shift of the curve in low *SIN3A* expression group, and no effect of *ESR1* expression on the curves was observed (Supplemental Fig. 6S). The relapse-free survival curves showed a clear lower shift with the reduced expression of the *SIN3A* mRNA in all patients, and the lower shift was identical to that seen for ER-positive breast cancers (Fig. 6E,F). *SIN3A* expression had no effect on the relapse-free survival curve for ER-negative breast cancers (Fig. 6G). These observations reveal that the reduction in the expression of *SIN3A* mRNA plays a role in the recurrence of ER-positive breast cancers.

## Discussion

In this report, we found 213 non-synonymous somatic mutations on the exon regions in 208 genes of DNAs extracted from breast cancers showing high expressions of ERα using different types of next-generation sequencers. Of the 213 somatic mutations, a novel mutation, *SIN3A* c.2830 C>T; p.Gln944*, caused an increase in *ESR1* mRNA expression and cell proliferation in MCF7 cells. The SIN3A mutant localized mainly in the cytoplasm apart from nuclear ERα in contrast to the nuclear localization of SIN3A-WT that co-localizes with ERα. The cytoplasmic localization attenuates the functions of SIN3A, leading to an increase in *ESR1* expression that accelerates the cell proliferation involved in the progression of breast cancers.

SIN3A has critical functions related to transcriptional regulation including nucleosome remodeling, DNA methylation, and N-acetyl-glucosamine transferase activity although it has no transcriptional activity by itself^22^. The functions are exclusively carried out by providing a platform for the assembly of transcriptional regulatory factors such as HDAC, MeCP2, Mad1, Elk-1, and p53 as a master scaffold^23^. As shown in Fig. 3A, the domain structures of SIN3A are composed of four PAH motifs, a HID present between PAH3 and PAH4, and a HCR in the C-terminal region. The domains in SIN3A are evolutionarily conserved in organisms from yeast to mammals. In this study, a novel somatic point mutation that changed a cytosine nucleotide into a thymine nucleotide at position of 2830 from the start codon of *SIN3A* (c.2830 C>T) was detected in a breast cancer tissue showing high expression levels of *ESR1*. The mutation is a nonsense mutation that changes a codon for glutamine into a stop codon (p.Gln944*) at position of 944 from that start methionine. SIN3A with p.Gln944* somatic mutation has a deleted C-terminal region including the C-terminal half of PAH4 and the entire HCR. In the C-terminal region, MeCP2 and O-linked N-acetylglucosamine transferase (OGT) and Alien can associate with SIN3A. MeCP2 that associates with the C-terminal region including the HCR of SIN3A mediates transcriptional repression through its interaction with methylated DNA^24^. OGT also inhibits transcriptional activities by introducing O-linked N-acetylglucosamine monosaccharides at the phosphorylation sites of transcription factors^25–28^. Alien functions as a co-repressor for selected members of the nuclear receptor superfamily such as vitamin D3 receptor and thyroid hormone receptor^29^. The assembly platform for these transcriptional repressors disappears in cells expressing SIN3A p.Gln944* due to its cytoplasmic localization, indicating that transcriptional activities may be dysregulated in cancer cells carrying the mutation. In this study, the expression of the *ESR1* mRNA was examined in cells transfected with *SIN3A* c.2830 C>T; p.Gln944* because Ellison-Zelski *et al*. have shown that SIN3A specifically represses *ESR1* expression at promoter regions in an estrogen-dependent manner. In practice, *ESR1* mRNA levels were significantly reduced in MCF7 cells transfected with *SIN3A*-WT as compared with those transfected with empty vector, and the expression was enhanced more than control level by transfection with *SIN3A* c.2830 C>T; p.Gln944* in the presence of 100 nM E2. The breast cancer tissue containing SIN3A p.Gln944* showed a 1.6-fold higher expression level of *ESR1* mRNA than breast cancer tissues containing SIN3A-WT.

Why does the C-terminal deletion mutant of SIN3A cause cell proliferation? SIN3A should exist in the nucleus in order to execute its transcriptional regulator activity; however, the SIN3A p.Gln944* mutant is also detected in the cytoplasm as shown in Fig. 3F. The nuclear localization of SIN3A without DNA binding activity may be governed by proteins that bind to its C-terminal region because both the C-terminal binding proteins MeCP2 and OGT possess nuclear localization signal^30,31^. SIN3A has a predicted nuclear localization signal (NLS) between the 2^nd^ and 3^rd^ PAH domains that may play a role in the nuclear transport of SIN3A (Supplementary Fig. 7S). The region around the NLS for SIN3A can bind to various proteins such as p53 and REST, which may mask NLS activity in the nucleus. On the other hand, a predicted nuclear export signal (NES) exists within the sequence from Leu902 to Ile909 of SIN3A, and this signal sequence might be exposed to the C-terminal region by the insertion of a stop codon at Gln944 in SIN3A c.2830 C>T (Supplementary Fig. 7S), although OGT in the region of amino acids 888–967 that includes the NES sequence associates with SIN3A-WT. The cytoplasmic transport of SIN3A c.2830 C>T; p.Gln944* may be mediated by the appearance of the NES sequence due to the deletion of the C-terminal binding region. The SIN3A mutant may localize in the cytoplasm accompanied by HDACs due to the conservation of the HDACs binding region. Endogenous SIN3A-WT is present in cells, but the factors that bind to SIN3A to inhibit transcription may be removed together with the SIN3A c.2830 C>T mutant; p.Gln944* when it moves to the cytoplasm, thus resulting in the loss of function. SIN3A inhibits the expression of *ESR1* mRNA through histone deacetylation in the near promoter region of *ESR1*^19^. The inhibitory pathway is blocked by the cytoplasmic localization, which leads to an increase in *ESR1* mRNA. The increased ERα trans-activates FOXK2 to repress the massive gene expression; however, the repression is also regulated by the interaction with the nuclear SIN3A complex. The interaction of FOXK2 may be prevented by the cytoplasmic localization of SIN3A p.Gln944*, and thus may cancel the growth inhibition of breast cancer cells^32^. Consistent with this report, cell proliferation was found to be rather suppressed in cells expressing SIN3A-WT, while SIN3A p.Gln944* localized in the cytoplasm, resulted in significantly enhanced cell proliferation. In the breast cancer tissue containing the SIN3A mutant, its distribution was mostly correlated with the tumor regions showing nuclear enlargement as observed in transcriptional activation, indicating that SIN3A mutant plays an important role in the progression of breast cancers. In the breast cancer tissue positive for *SIN3A* c.2830 C>T, the SIN3A protein appeared to be decreased as compared with *SIN3A*-WT (Fig. 6A-D). The low expression of *SIN3A* shifts in the lower direction in the relapse-free survival curve of ER-positive breast cancer (Fig. 6F), indicating that the decrease in the amount of *SIN3A* mRNA accelerates the recurrence of ER-positive breast cancers. The low expression of *SIN3A* mRNA also decreases survival rates in non-small cell lung cancer and renal cell carcinomas^33^. In a previous report, *SIN3A*-knock down cells showed a significant increase in invasive activity, leading to an increased rate of metastasis^34^. Metastasis to other organs may be involved in the breast cancer relapse.

Mutations in *SIN3A* have previously been reported to cause mild intellectual disabilities via the reduction of cortical neurogenesis in the brain^35^. Most mutations are nonsense point mutations that lead to the decay of the mRNA product or to large deletions of the genome that includes the whole *SIN3A* gene, and share the common feature of the absence of the C-terminal region of SIN3A. These observations show that the C-terminal region of SIN3A, including the HCR, plays a critical role in pathogenesis.

This is the first study to describe the novel somatic mutation of *SIN3A* c.2830 C>T; p.Gln944* that causes the deletion of the C-terminal region from Gln944 and contributes to cell proliferation in breast cancer. The transcriptional regulation of SIN3A in luminal subtype breast cancers would provide a new therapeutic target for breast cancers.

## Materials and Methods

### Materials

Anti-SIN3A mouse monoclonal antibody (sc-5299) and *ESR1* siRNA (human, sc-29305) were purchased from Santa Cruz (CA). Plasmids pFN21A (G2821) and pMACS4.1 were purchased from Promega (Madison, WI) and (Miltenyi Biotec., Bergisch Gladbach, Germany), respectively. An expression vector of *halo*-tagged *SIN3A* was purchased from Kazusa cDNA/ORF clone collection (FHC11647). pEGFP-C1-ERα was a gift from Michael Mancini (Addgene plasmid #28230)^36^. All other reagents were commercially available.

### Clinical samples

The informed consent regarding the use of samples were received from all patients, and the protocol was approved by the Local Ethics Committee of Yamaguchi University School of Medicine. All the experiments were carried out in accordance with the guideline and regulations from Yamaguchi University. Breast tumor samples were obtained from patients ranging in age from 35 to 84 years (58.2 ± 12.6, mean ± SD, n = 16) who underwent surgery to remove the tumors. The samples were confirmed pathologically to be tumors^37,38^. The tumor samples were divided sequentially in the operating room, and representative segments were excised and preserved at −80 °C until processing. Whole blood cells were collected from patients prior to surgery.

### Measurements of mRNAs for conventional biomarkers in breast cancer tissues

mRNA measurements were carried out as described previously^39^. Briefly, the frozen breast cancer tissues were minced, placed into a tube containing beads, and homogenized in 9 volumes of lysis buffer with a Retsch MM300 (Qiagen, Hilden, Germany) mixer mill. After the removal of the beads, the samples were centrifuged at 12,000 xg for 20 min at 4 °C, and the supernatants were immediately used for total RNA extraction. Total RNA was isolated using the RNeasy mini kit (Qiagen) as described previously^40^. Five hundred nanograms of total RNA were added to mixtures of 2.5 µM random primer 9 (NewEngland Biolabs, Beverly, MA), 500 µM dNTPs, 40 U RNase inhibitor (Takara Bio. Inc., Shiga, Japan) and 10 U M-MuLV reverse transcriptase (NewEngland Biolabs), and incubated at 42 °C for 60 min and then at 90 °C for 10 min. The mRNA expression values of *ESR1*, *PGR*, and *HER2* were measured by a Quanti Tect SYBR Green PCR kit (Qiagen) with a Rotor-gene 6000 (Qiagen) at 95 °C for 15 min, followed by 45 cycles of 94 °C for 15 sec, 55 °C for 30 sec and 72 °C for 30 sec. The primers used for quantitative PCR (qPCR) are shown in Supplementary Table 10S. The calculated data were normalized to the values for the *GAPDH* used as a control gene.

### Library preparation for WES by SOLiD5500

Genomic DNAs were extracted from the tumors and whole blood cells using a PureLink Genomic DNA mini kit (Thermo Fisher Scientific, MA) according to the manufacturer’s instructions. The genomic DNAs were used for the preparation of a fragment library by the 5500 SOLiD fragment library core kit (Thermo Fisher Scientific). Briefly, 1 µg or 3 µg of genomic DNA was sheared using the Covaris^®^ S220 System (Covaris Inc, MA) for 60 sec with a duty cycle of 10%, 6 cycles, 100 cycles/burst at 5 °C. The shearing DNA was repaired at the ends, and the DNA was purified using agencourt AMPure^®^ XP reagent (BeckmanCaulter, CA) to prepare DNA fragments with a size range of 100–300 bp. The adaptors were ligated to both ends of the DNA to generate a fragment library. The fragment library was purified by agencourt AMPure^®^ XP reagent. The purified library was amplified by PCR using Platinum PCR amplification mix. PCR was performed at 95 °C for 5 min, 6 cycles of 95 °C for 15 sec, 62 °C for 15 sec and 70 °C for 1 min, and 70 °C for 5 min. The amplified fragment library was purified, and the concentration was measured by qPCR with TaqMan probes. The length of the amplified fragment library was confirmed on an Agilent 2100 Bioanalyzer (Agilent, CA) using a high-sensitivity DNA kit (Agilent). The size of the DNA fragments ranged from 200 bp to 400 bp with a mean size of 270 bp.

### WES analysis by SOLiD5500

The enrichment of the library including exon regions was performed using the TargetSeq™ exome enrichment system (Thermo Fisher Scientific) according to the manufacturer’s protocol. Briefly, 500 ng of the fragment library was added into a 1.5-ml tube containing reaction mixture including 0.5 µl human *Cot-1* DNA and 1 nmol TargetSeq™ blockers. The mixture was dried with a vacuum concentrator at 60 °C, and then the mixture including 7.5 µl of TargetSeq™ hybridization solution A and 3 µl of TargetSeq™ hybridization enhancer B was added into the tube including the dried sample. The sample was denatured by incubating at 95 °C for 10 min, and then combined with 4.5 µl TargetSeq™ exome probe pool including biotinylated oligos ranging in size from 50 to 120 bases. The reaction mixture was incubated on a thermal cycler at 47 °C for 72 hrs. The hybridized DNA was isolated using streptavidin-coated magnetic beads, and then amplified by PCR. PCR was performed at 95 °C for 5 min, 10 cycles of 95 °C for 15 sec, 62 °C for 15 sec and 70 °C for 1 min, and 70 °C for 5 min. The amplified DNA was purified by agencourt AMPure^®^ XP reagent, and the concentration was determined by quantitative PCR with TaqMan probes. The length of the hybridized DNA was confirmed with the Agilent 2100 bioanalyzer using a high-sensitivity DNA kit. The size of the DNA ranged from 200 bp to 400 bp, with an average of fragment size of approximately 270 bp. The enrichment of the exon regions was confirmed by qPCR using primer pairs for 4 genes (*RUNX2*, *PRKG1*, *SMG1*, *PLAU*) as positive controls and 2 genes (*PLAU-3*′*UTR*, *PLAU-Promoter*) as negative controls. The qPCR of the regions was performed with a Rotor-Gene 6000 (Qiagen) using a QuantiTect SYBR Green Kit (Qiagen) in the presence of the following primers: forward primer 5′-CGC ATT CCT CAT CCC AGT ATG-3′ and reverse primer 5′-AAA GGA CTT GGT GCA GAG TTC AG-3′ for *RUNX2*, forward primer 5′-CCC ACC GCC TTC GAC AT-3′ and reverse primer 5′-CCT GCT TAC TGT GGG CTC TTG-3′ for *PRKG1*, forward primer 5′-CTC GCT TAA CCA GAC TCA TCT ACT GT-3′ and reverse primer 5′-ACT TGG CTC AGC TGT ATG AAG GT-3′ for *SMG1*, forward primer 5′-GTG GCC AAA AGA CTC TGA GG-3′ and reverse primer 5′-CCT CCA CAC ACG TAG GTG AC-3′ for *PLAU*, forward primer 5′-CAA ATC TCC CTG GTG CTT GT-3′ and reverse primer 5′-CCT GCC CTA CAG CTC TCC TA-3′ for *PLAU-3*′*UTR*, forward primer 5′-AGC TGG GCG AGG TAG AGA GT-3′ and reverse primer 5′-CAG CGT CTG GAC TGA GGA AT-3′ for *PLAU-Promoter*. The ΔCT for the six loci was calculated by subtracting the CT value of the exon-enriched DNA library from the CT value of the unenriched DNA library, and the DNA libraries concentrated more than 48% in the exon regions were used for emulsion PCR reaction. After emulsion PCR was performed using 0.7 pM of DNA library, the emulsions were broken and the beads attaching the amplified DNA were collected. Approximately 280 million beads per lane were applied to the flowchip in a SOLiD5500 (Thermo Fisher Scientific)^41^.

### Detection of somatic mutations in breast cancers with high expression of ERα

The raw data of the XSQ format obtained from the sequencer were converted to a csfasta file and a quality file by XSQTools (Thermo Fisher Scientific). Reads with more than 2 ambiguous nucleotides and reads with quality scores less than 20 as calculated by the Phred program were removed using CLC Genomics Workbench software (ver.8.01, Qiagen). Long reads with more than 1000 nucleotides and short reads with fewer than 20 nucleotides were also discarded. The trimmed reads were mapped to the NCBI human reference genome hg19 in default setting. Briefly, the reads were aligned to reference using the setting conditions with mismatch cost of 2, insertion cost of 3, and deletion cost of 3. In addition, the reads were mapped when at least half of the alignment matched the reference sequence (length fraction of 0.5), and the matched alignment was at least 80% identical to the reference sequences (similarity fraction of 0.8), and non-specifically matched reads were mapped randomly. To detect a point mutation, the length fraction was changed to 1.0 and the similarity fraction to 0.98. Using the mapping files, single nucleotide variants (SNVs) were detected using CLC Genomics Workbench. Briefly, SNVs were detected according to the following criteria: coverage at the genomic position more than 5; base quality of variant position higher than 20; at least 2% of the total reads detected as a variant; and more than 4 reads observed as a variant. The annotation of detected variants was analyzed using CLC Genomics workbench software. To detect non-synonymous somatic mutations, the synonymous variants were removed, and the known SNVs in dbSNP common database (ver.150) and the variants detected by WES in blood samples were also removed. In cases for which blood samples were unavailable, the somatic mutations were determined by subtraction of the data for the other whole blood samples used in this study and the variant database of dbSNP (common, ver.150). The remaining variants were annotated with the gene names listed in the TargetSeq BED file (Thermo Fisher Scientific).

### Selection of ERα-associated genes from somatic mutations of breast cancers

The ERα-associated genes were selected from the somatic mutations detected in breast cancers using IPA (Qiagen)^42^. The lists of gene groups associated with ERα (gene symbol *ESR1*) were produced using the SEARCH function of the Ingenuity Knowledge Base containing approximately 5 million data sets extracted from scientific publications and databases. Based on the lists, the network between the genes with the somatic mutations and ERα-associated genes was reconstituted using the GROW function of the BUILT menu in the IPA software. The *ESR1* gene was entered as GROW molecules. Both direct and indirect interactions for interaction were selected, and both upstream and downstream molecules for molecules were entered. The search molecules were limited to the list of somatic mutations in this study. For other parameters, the default settings were used in the IPA pathway search.

### Confirmation of somatic mutations in selected ERα-associated genes by target re-sequencing

To confirm the mutations, 359 regions including the point mutation in the ERα-associated genes were amplified by multiplex PCR using primer sets designed with an Ion AmpliSeq Designer (http://ampliseq.com). The sequences of the amplicons were analyzed by the Ion PGM system. Briefly, 10 ng of genomic DNA was amplified by 17 cycles of PCR at 99 °C for 15 sec and at 60 °C for 4 min after incubation at 99 °C for 2 min using the Ion AmpliSeq HiFi in the Ion AmpliSeq Library kit 2.0 (Thermo Fisher Scientific) with primer sets as shown in Supplementary Table 2S. The PCR products were incubated with 2 µl of FuPa reagent to partially digest the primer sequences at 50 °C for 10 min, 55 °C for 10 min and 60 °C for 20 min, and then were ligated to P1 adaptor and barcode adaptors from the IonXpress Barcode adaptors 1–16 kit (Thermo Fisher Scientific). After ligation, the libraries were purified by agencourt AMPure^®^ XP reagent, and amplified by PCR using Platinum^®^ PCR supermix high fidelity mix (Thermo Fisher Scientific) at 98 °C for 2 min, 5 cycles of 98 °C for 15 sec and 60 °C for 1 min. The concentration of the amplified library was quantified by qPCR using a QuantiTect SYBR Green Kit and the length was measured with an Agilent 2100 Bioanalyzer using a high-sensitivity DNA kit. The size of the libraries ranged from 250 bp to 360 bp with a mean fragment size of approximately 320 bp. After quantification, the libraries were diluted to 20 pM, and then equal volumes of the libraries were combined. The library mixture was amplified by emulsion PCR and loaded on an Ion 318 Chip v2 BC (Thermo Fisher Scientific) using Ion Chef system (Thermo Fisher Scientific). The DNAs on the chip were analyzed with an Ion PGM IC sequencing reagent kit (Thermo Fisher Scientific) using the Ion PGM sequencer.

The raw data of the sequences were converted to sequence reads, and adaptor sequences and low quality bases were removed using Torrent suite software (ver. 4.2.1). The processed reads were aligned to the NCBI human reference genome hg19 using a Torrent mapping alignment program. The variants were detected under the following conditions: more than 20 for coverage, higher than 10 for read quality, at least 2% of variants for the total reads, and more than 4 of the variant coverage on either strand using Torrent variant caller plugin software.

### Confirmation of a somatic mutation in *SIN3A* by Sanger sequencing

Genome DNAs extracted from breast cancer tissues were amplified by PCR using primers 5′-TGCGTCCACAGTACCAACC-3′ and 5′-ATTTGTTCCCAAGCCGAACG-3′ for the region from 75684340 to 75684709 on chromosome 15 including the *SIN3A* mutation. The PCR products were incubated at 37 °C for 20 min in a mixture of 5 U of exonuclease I (NewEngland Biolabs) and shrimp alkaline phosphatase (TaKaRa Bio. Inc.), and were purified using a BigDye^®^Xterminator™ purification kit (Thermo Fisher Scientific). The purified PCR products were sequenced using a BigDye^®^Terminator v3.1 cycle sequencing kit (Thermo Fisher Scientific) and analyzed on a 3130xl genetic analyzer (Thermo Fisher Scientific)^43^.

### Expression of a somatic mutation of *SIN3A* in MCF7 cells

The expression vector for the somatic mutation *SIN3A* c.2830 C>T; p.Gln944* was produced by introduction into the *SIN3A*-WT by site-directed mutagenesis as described previously^44^. Briefly, the expression vector of *halo*-tagged *SIN3A* was methylated with CpG methyltransferase (NewEngland Biolabs) in the presence of S-adenosylmethionine (NewEngland Biolabs), and then amplified by PCR using Taq polymerase (KOD Plus Neo, Toyobo, Osaka, Japan) with primers for mutagenesis as shown below at 95 °C for 2 min, 20 cycles of 98 °C for 10 sec and 68 °C for 5 min. Primers used for mutagenesis were 5′-AGAGTGACAGCCCTGCCATTTAGCTACGTCT-3′ and 5′-AATGGCAGGGCTGTCACTCTTGTCTCGCTT-3′. The PCR products were selectively amplified in *DH5*α strain *Escherichia coli*. The point mutation was confirmed by Sanger sequencing. MCF7 cells were cultured in DMEM medium supplemented with 10% heat-inactivated FBS, 100 U/ml of penicillin and 100 mg/ml of streptomycin in a humidified atmosphere of 5% CO_2_ in air at 37 °C. Total 1 µg of the indicated vectors with pMACS 4.1 truncated CD4 expression and pGFP vector was added to 10 µl of solution R in a Neon transfection kit (Thermo Fisher Scientific) containing 1.0 × 10^6^ of MCF7 cells. The expression vectors were introduced into MCF7 cells with a Neon electroporator (Thermo Fisher Scientific) under the experimental conditions of 2 pulses with a width of 20 and voltage at 1250, and subsequently seeded on a culture plate. The cells were incubated with anti-CD4 antibody magnet beads (Miltenyi Biotec.) after the dissociation with phosphate-buffered saline (PBS) with EDTA buffer, and were separated with an autoMACS pro (Miltenyi Biotec.) after 24 hrs of transfection.

### Measurement of *ESR1* mRNA in SIN3A p.Gln944*-expressed MCF7 cells

Total RNA was isolated from MCF7 cells transfected with the indicated vectors using Fast Gene RNA Basic kit (Nippon Genetics, Tokyo, Japan). One hundred nanograms of total RNA were added to a mixture of 250 nM Oligo dT primer (Toyobo), 500 µM dNTPs, 40 U RNase inhibitor (Takara Bio. Inc.) and 10 U M-MuLV reverse transcriptase (NewEngland Biolabs), and incubated at 42 °C for 60 min and 90 °C for 10 min. The expressions of *ESR1* mRNA were measured using the synthesized DNAs as templates by a Quanti Tect SYBR Green PCR kit (Qiagen) with a Rotor-gene 6000 (Qiagen) at 95 °C for 15 min, followed by 45 cycles of 94 °C for 15 sec, 55 °C for 30 sec and 72 °C for 30 sec. The primers used for qPCR are shown in Supplementary Table 10S. The calculated data were normalized to the values for *GAPDH* used as a control gene.

### Intracellular localization of SIN3A p.Gln944* in MCF7 cells

The transfected MCF7 cells were incubated with culture medium containing 5 µM TMR ligand (Promega) at 37 °C for 15 min. The culture medium was replaced with fresh culture medium, and the cells were incubated at 37 °C for 30 min. After washes with PBS, the cells were incubated with culture medium containing 40 µg/ml Hoechst33342 at 37 °C for 15 min. The cells were then washed with PBS, and the culture medium was replaced with fresh culture medium. The stained cells were imaged with a confocal microscope LSM710 (Zeiss, Oberkochen, Germany). The localizations of SIN3A were analyzed using IN Cell analyzer 2000 (GE Healthcare, Buckingamshire, England). The cells were seeded in a 96-well microplate (µClear plate, Greiner Bio-one), and stained with Hoechst33342 for the nucleus and fluorescence-labeled Halo-tag ligand TMR for Halo-SIN3A. The cells were excited at 355 nm for Hoechst33342 and at 543 nm for the TMR ligand, and images were photographed with laser autofocus mode using a 20x objective lens (Nikon, Tokyo, Japan) in an IN Cell Analyzer 2000. The exposure time was 0.1 sec for Hoechst33342 and 0.3 sec for the TMR ligand. The photographs were analyzed using IN Cell workstation software (GE Healthcare) or IN Carta software (GE Healthcare). For IN Cell workstation software, the multi target analysis module was used, and the algorithm for the nuclear region was applied to the staining of Hoechst33342 and the algorithm for the cell region was used for Halo-SIN3A staining. The total signal intensities in each region were calculated by subtracting the background signal intensities. For IN Carta software, mononucleated cells were selected from the application, and nuclei and cell as target types were applied to the determination of signal intensities in each region. The total signal intensities in each region were calculated by subtracting the background intensities.

### Cell proliferation assay of MCF7 cells transfected with *SIN3A* c.2830 C>T

MCF7 cells transfected with *halo*-tagged empty vector, *halo*-*SIN3A*-WT, or *halo*-*SIN3A* c.2830 C>T with pMACS 4.1, and were incubated with anti-CD4 antibody magnet beads, and were separated with the autoMACS pro after 24 hrs of transfection. The cells were seeded onto an E-plate, and the cell impedances were monitored every 15 min for 24 hrs with a xCELLigence RTCA DP instrument (ACEC Biosciences, CA) in the presence of 100 nM E2.

### Immunohistochemical analysis of breast cancer tissues with *SIN3A* p.Gln944*

The tissues were fixed with 4% paraformaldehyde in PBS for 12 hrs at room temperature. After washing with PBS, the tissues were embedded in paraffin blocks, and cut into 4 µm thick tissue sections using a microtome. The tissue sections were deparaffinized in xylene, and stained with hematoxylin and eosin (HE). For immunohistochemical staining, the sections were treated with Target retrieval solution (Agilent) to activate the antigens and with 0.3% H_2_O_2_ at room temperature for 10 min, and then incubated with anti-SIN3A antibody for the N-terminal region of SIN3A at 4 °C overnight. The samples were visualized with immunoperoxidase polymer reagent conjugated Fab fragments for anti-mouse antibody (MAX-PO, Nichirei Bioscience, Inc., Tokyo, Japan). After staining with anti-SIN3A antibody, the sections were weakly counterstained with hematoxylin to detect the nuclei.

### Survival curves for breast cancer patients according to *SIN3A* mRNA expression

Overall and relapse-free survival curves were drawn based on the expression of *SIN3A* mRNA using a Kaplan-Meier Plotter (http://kmplot.com/analysis)^45^. The classification of *ESR1* expression was determined from the gene expression data. To produce survival curves based on gene expression, the *SIN3A* (238005_s_at) probe as a gene symbol was selected and the auto-select cutoff was used. P values of the Kaplan-Meier Plots were calculated using a log-rank test, and hazard ratios with 95% confidence intervals were determined from the survival rates in each group.

### Detection of SIN3A protein in MCF7 cells by SDS-PAGE and Western blotting

The cells were sonicated in 4 volumes of lysis buffer [1% Triton X-100, 20 mM Tris (pH 7.5), 150 mM NaCl, 1 mM EDTA, 1 mM EGTA, 2.5 mM sodium pyrophosphate, 1 mM ß-glycerol phosphate, 1 mM sodium orthovanadate, 1 µg/ml leupeptin, 1 mM phenylmethanesulfonyl fluoride], and centrifuged at 12,000 xg for 20 min at 4 °C. The supernatants were collected and were incubated with Halo-tag TMR ligand for 15 min. Electrophoresis and western blotting were carried out after the addition of SDS sample buffer as described previously^39^. Briefly, the extracts and molecular mass standards were electrophoresed in 10% (w/v) polyacrylamide gels in the presence of SDS, and the fluorescence of TMR ligand on the gels was detected with a Typhoon FLA 9000 (GE Healthcare). The gels were transferred to nitrocellulose membranes. The blots were blocked with 5% non-fat dry milk in Tris-buffered saline containing 0.05% (w/v) Tween-20, and incubated with anti-Halo-tag antibody (Promega), anti-SIN3A antibody (SantaCruz), anti-GFP antibody conjugated HRP (Nacalai tesque, Kyoto, Japan) or anti-α-Actin antibody (Sigma-Aldrich). The blots were then washed, and the antigens were visualized by enhanced chemiluminescence detection reagents, and observed under a Amarsham Imager (GE Healthcare). The densities of the detected bands were measured by a ImageQuant TL software (GE Healthcare). The sample concentrations were prepared based on protein contents of α-Actin.

## Electronic supplementary material

Supplementary Information

Supplementary Table1S

Supplementary Table2S

Supplementary Table3S

Supplementary Table4S

Supplementary Table5S

Supplementary Table6S

Supplementary Table7S

Supplementary Table8S

Supplementary Table9S

Supplementary Table10S
