# Supplementary material for: A novel somatic mutation of SIN3A detected in breast cancer by whole-exome sequencing enhances cell proliferation through ERα expression
Source: Sci Rep. 2018 Oct 30;8:16000. doi: 10.1038/s41598-018-34290-1 (PMC6207735; doi:10.1038/s41598-018-34290-1)
Supplement: Supplementary file 1 — Supplementary Information [file 41598_2018_34290_MOESM1_ESM.pdf]

A novel somatic mutation of SIN3A detected in breast cancer by whole-exome sequencing enhances cell proliferation through ERα expression

Short title: A novel mutation of SIN3A in breast cancer

Kenji Watanabe[1], Shigeru Yamamoto[2], Syuiti Sakaguti[3], Keishiro Isayama[4], Masaaki Oka[2], Hiroaki Nagano[2], Yoichi Mizukami[1]*

From the [1]Institute of Gene Research, Yamaguchi University Science Research Center, Yamaguchi, 755-8505, Japan, [2]Department of Gastroenterological, Breast and Endocrine Surgery, Yamaguchi University Graduate School of Medicine, Yamaguchi, 755-8505, Japan, [3]Institute of Radioisotope Research and Education, Yamaguchi University Science Research Center, Yamaguchi, 755-8505, Japan, [4]Institute of Life Science, Yamaguchi University Science Research Center, Yamaguchi, 755-8505, Japan

*Correspondence to: Yoichi Mizukami

The Institute of Gene Research, Yamaguchi University Science Research Center

1-1-1 Minami-Kogushi, Ube, Yamaguchi 755-8505, Japan

Tel&FAX: +81-836-22-2183

E-mail: **mizukami@yamaguchi-u.ac.jp**

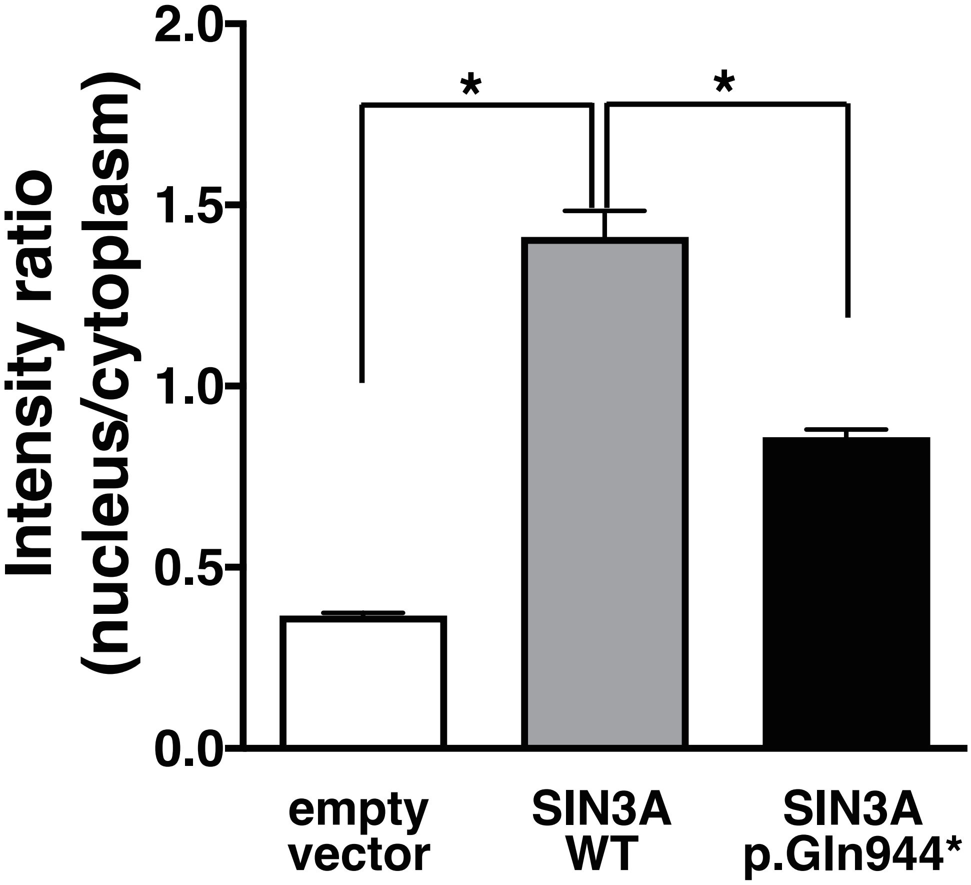

Supplementary Fig.1S

Quantitative analysis of intracellular localizations of SIN3A-WT and SIN3A p.Gln944*

Intracellular localizations of SIN3A-WT or SIN3A p.Gln944* were calculated from the total fluorescence intensities in the nucleus and cytoplasm using an IN Cell Workstation software. The data represent the means ± SE obtained from 2757 cells transfected with empty vector, 1833 cells transfected with SIN3A-WT, and 2206 cells transfected with SIN3A c.2830C>T. Statistical significances were determined by ANOVA with the Tukey–Kramer multiple comparison test (*P < 0.05).

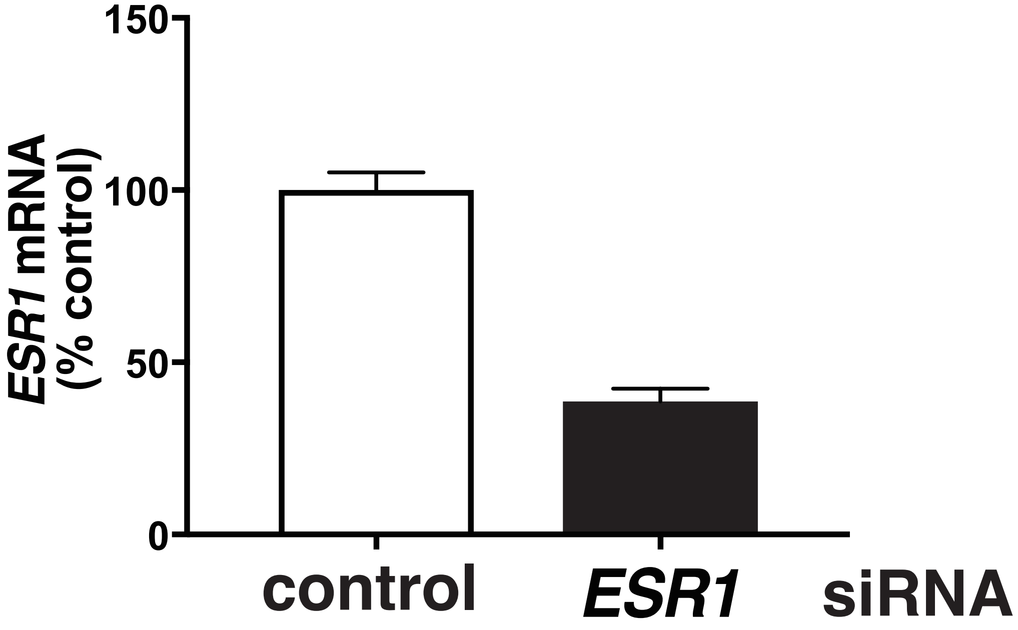

Supplementary Fig.2S

Expression of ESR1 mRNA in MCF7 cells transfected with siRNA for ESR1

MCF7 cells were transfected with control siRNA or siRNA for ESR1. After transfection, the cells were cultured in culture medium for 24 hrs. Total RNA was extracted and converted to cDNA by reverse transcriptase. The expressions of ESR1 mRNA were determined by qPCR using the primers indicated in Supplemental Table10S. The data represent the means ± SE, and were obtained from 3 independent experiments.

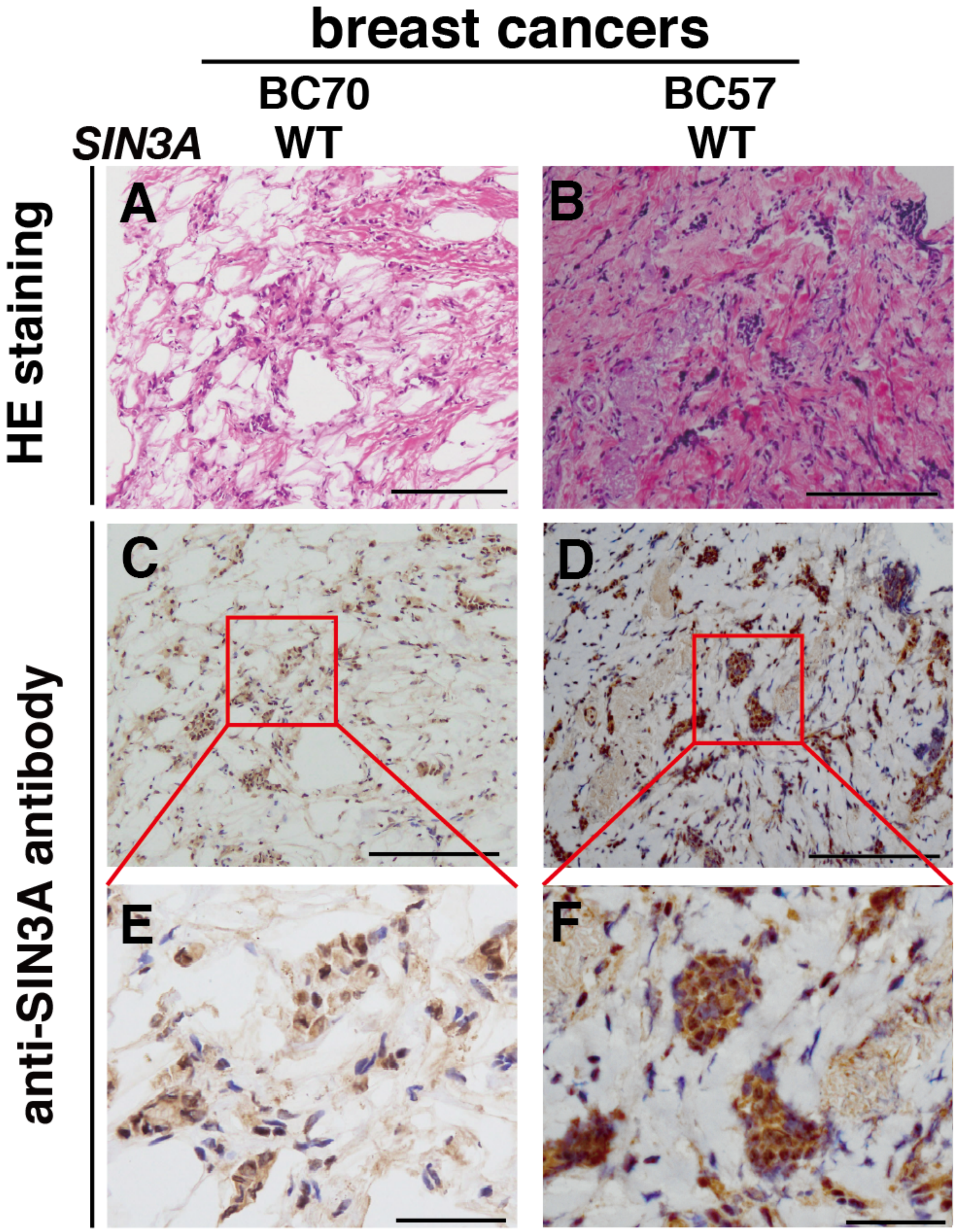

Supplementary Fig.3S

Intracellular distribution of SIN3A in breast cancer tissues

Tissues with SIN3A-WT were fixed with 4% paraformaldehyde, and cut into 4 μm thick sections after embedding in paraffin blocks. The sections were stained with hematoxylin and eosin (A and B), and then stained with anti-SIN3A antibody for the N-terminal region concurrently with counterstaining with hematoxylin to detect the nuclei (C-F). The boxed regions in the pictures stained with anti-SIN3A antibody (C and D) are magnified 4-fold (E and F). The scale bars are 200 μm (A-D) and 50 μm (E and F).

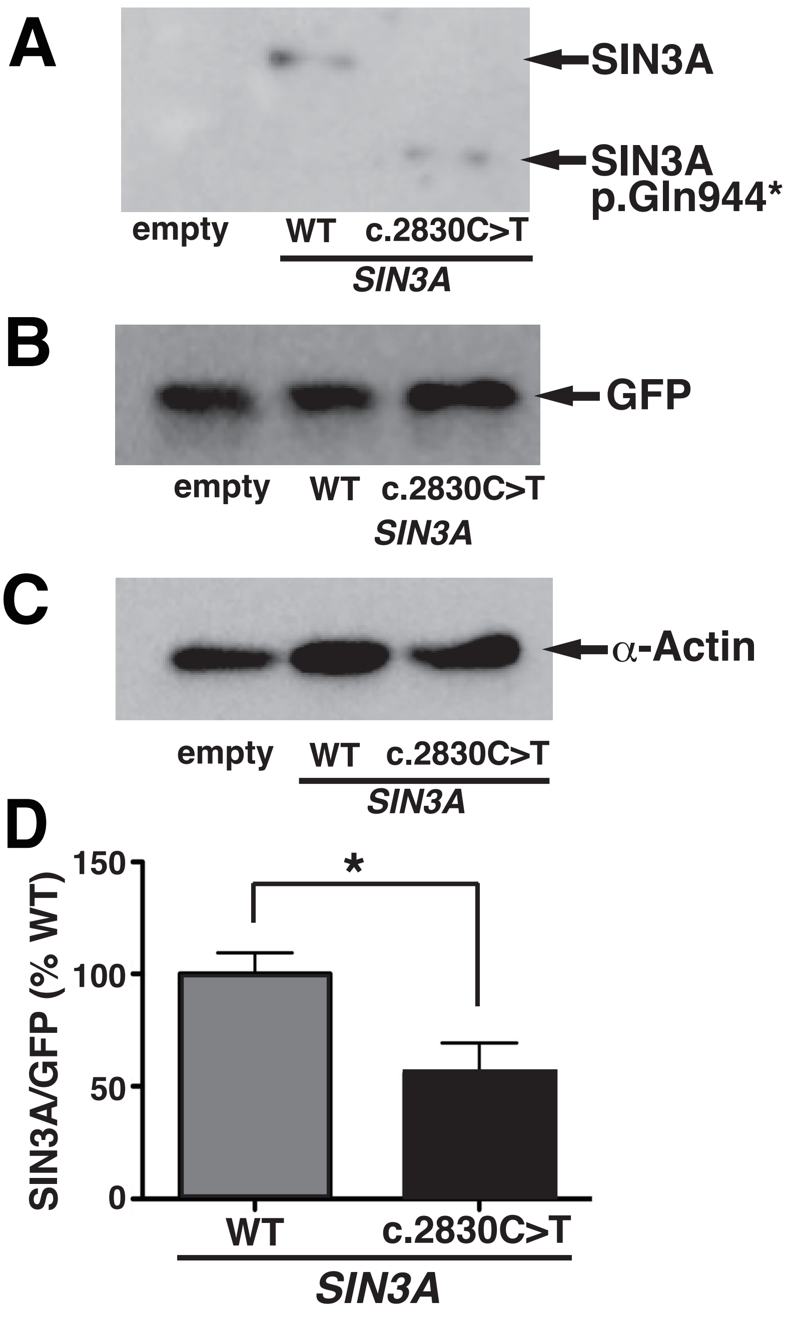

Supplementary Fig.4S

Detection of SIN3A proteins in MCF7 cells transfected with SIN3A-WT and c.2830C>T mutant by Western blotting

(A-D) MCF7 cells were transfected with SIN3A-WT, SIN3A c.2830C>T, or empty vector together with pMACS4.1 and pGFP, and the cells were cultured in culture medium for 24 hrs. The vector-transfected cells were separated with anti-CD4 antibody magnet beads. After the extraction from the cells, the extracts were subjected to immunoblotting using anti-SIN3A antibody (A), anti-GFP antibody (B) or anti-α-Actin antibody (C). The bands corresponded to the indicated proteins were analyzed by Image Quant QL (D). The figure shows representative data obtained from 3 independent experiments. The data represent the means ± SE. Statistical significances were determined by two-tailed Student' s t test (*$P < 0.05$).

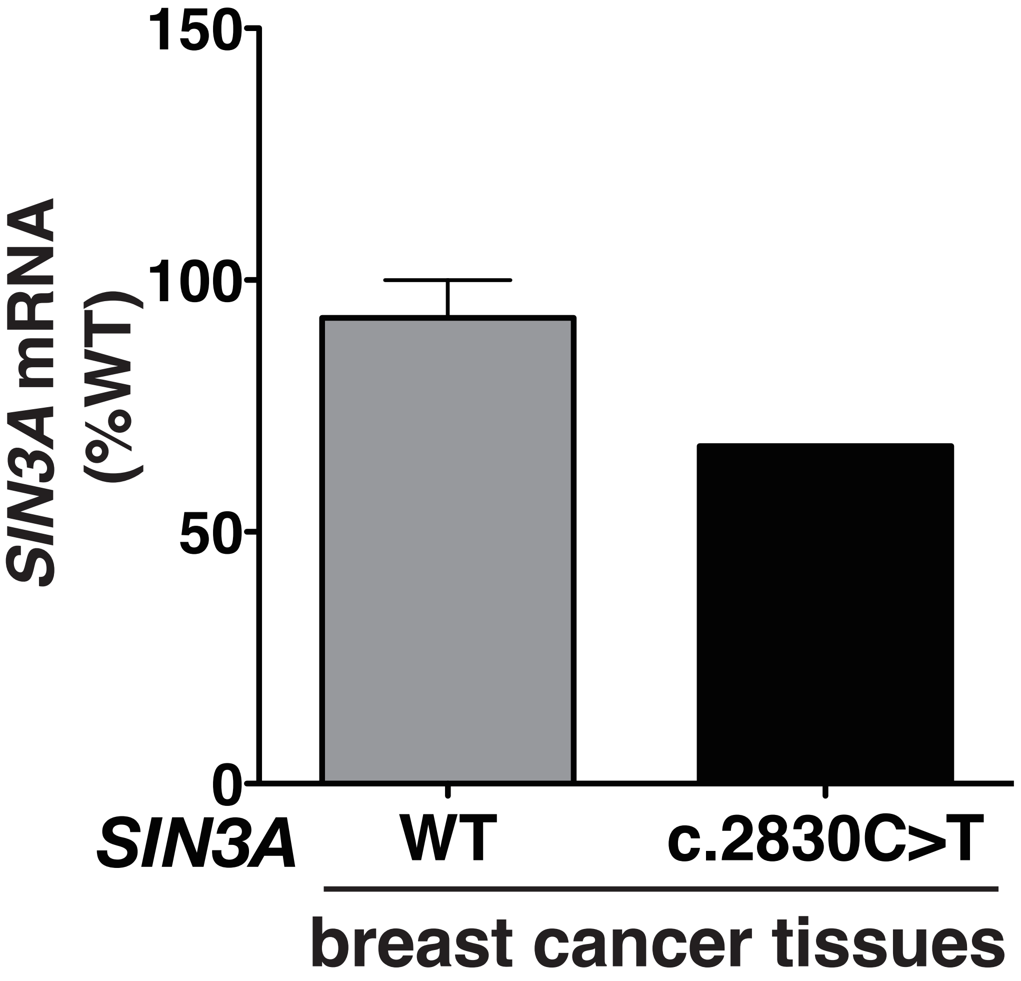

Supplementary Fig.5S

SIN3A mRNA expression in breast cancer tissues.

Total RNAs were extracted from tissues with SIN3A-WT (n=3) or a tissue with SIN3A c.2830C>T (n=1), and converted to cDNA by reverse transcriptase. The expressions of SIN3A mRNA were determined by quantitative PCR using the primers for SIN3A. The mRNA expressions were normalized to the expression of GAPDH. The data in SIN3A-WT represents the means ± SE.

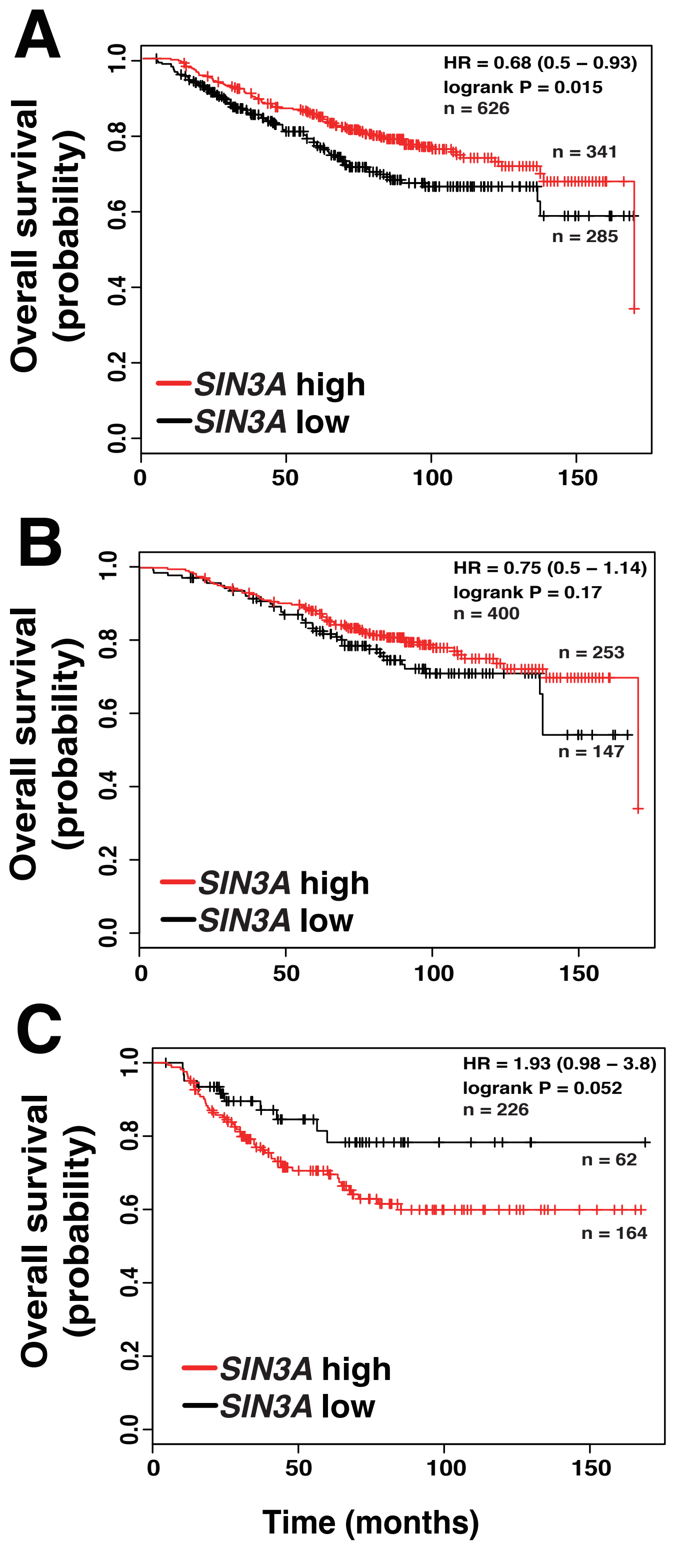

Supplementary Fig.6S

Overall survival curves of patients with breast cancer according to SIN3A mRNA expression

Overall survival curves were drawn for the high SIN3A expression group (red) and low SIN3A expression group (black) for all (A, n=626), ER-positive (B, n=400), or ER-negative (C, n=226) breast cancer patients using the Kaplan-Meier Plotter.

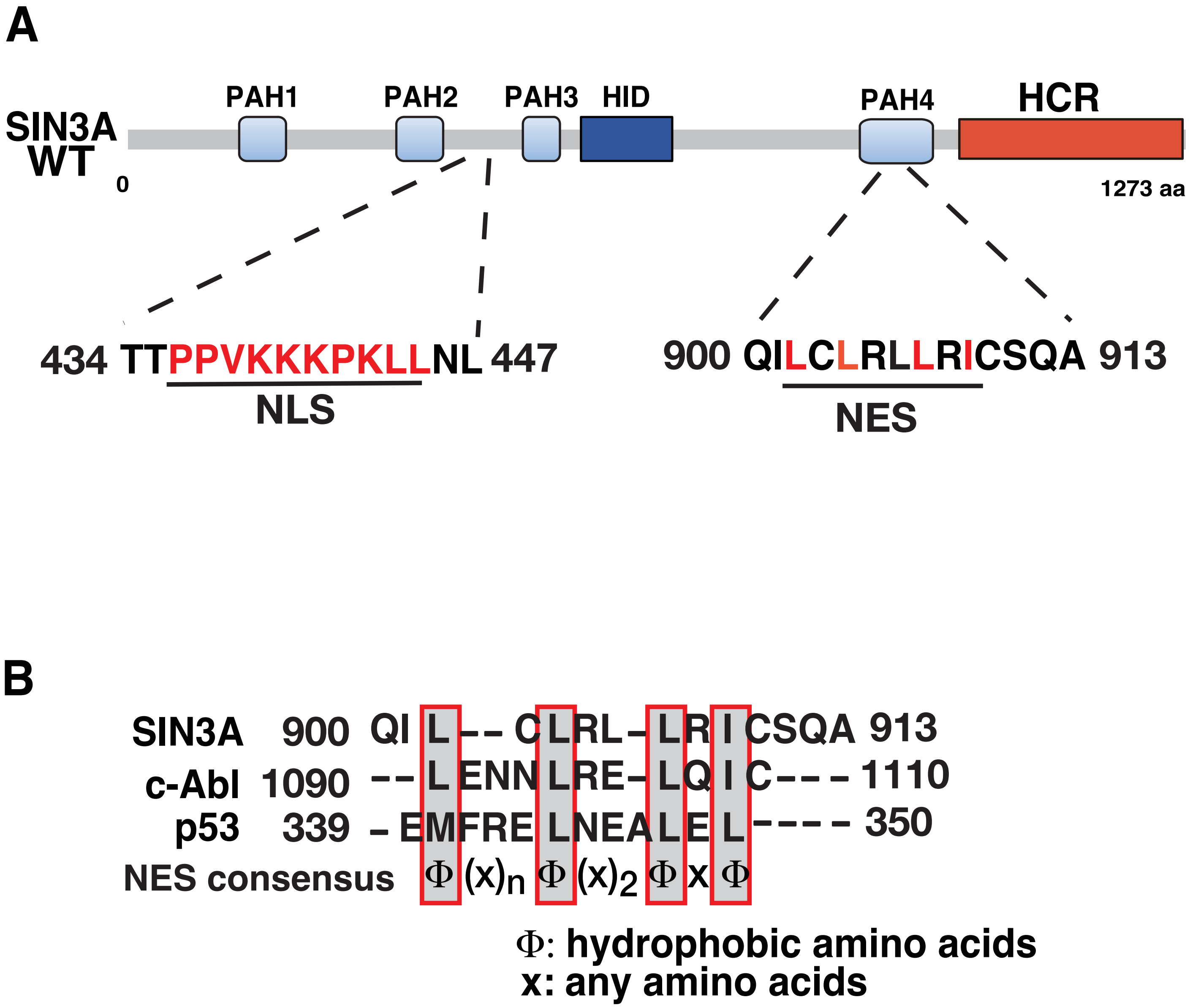

Supplementary Fig.7S

Nuclear localization signal and nuclear export signal in the molecular structure of SIN3A.

(A) The predicted nuclear localization signal (NLS) and the predicted nuclear export signal (NES) in the molecular structure of SIN3A are shown. SIN3A has the predicted NLS sequence at positions 434 to 447 of the amino acid sequence, and the predicted NES sequence at positions 900 to 913 of the amino acid sequence. (B) The consensus sequence of NES is shown. c-Abl and p53 are known to have a NES sequence in their molecular structures, and the sequence in SIN3A is consistent with the known NES.

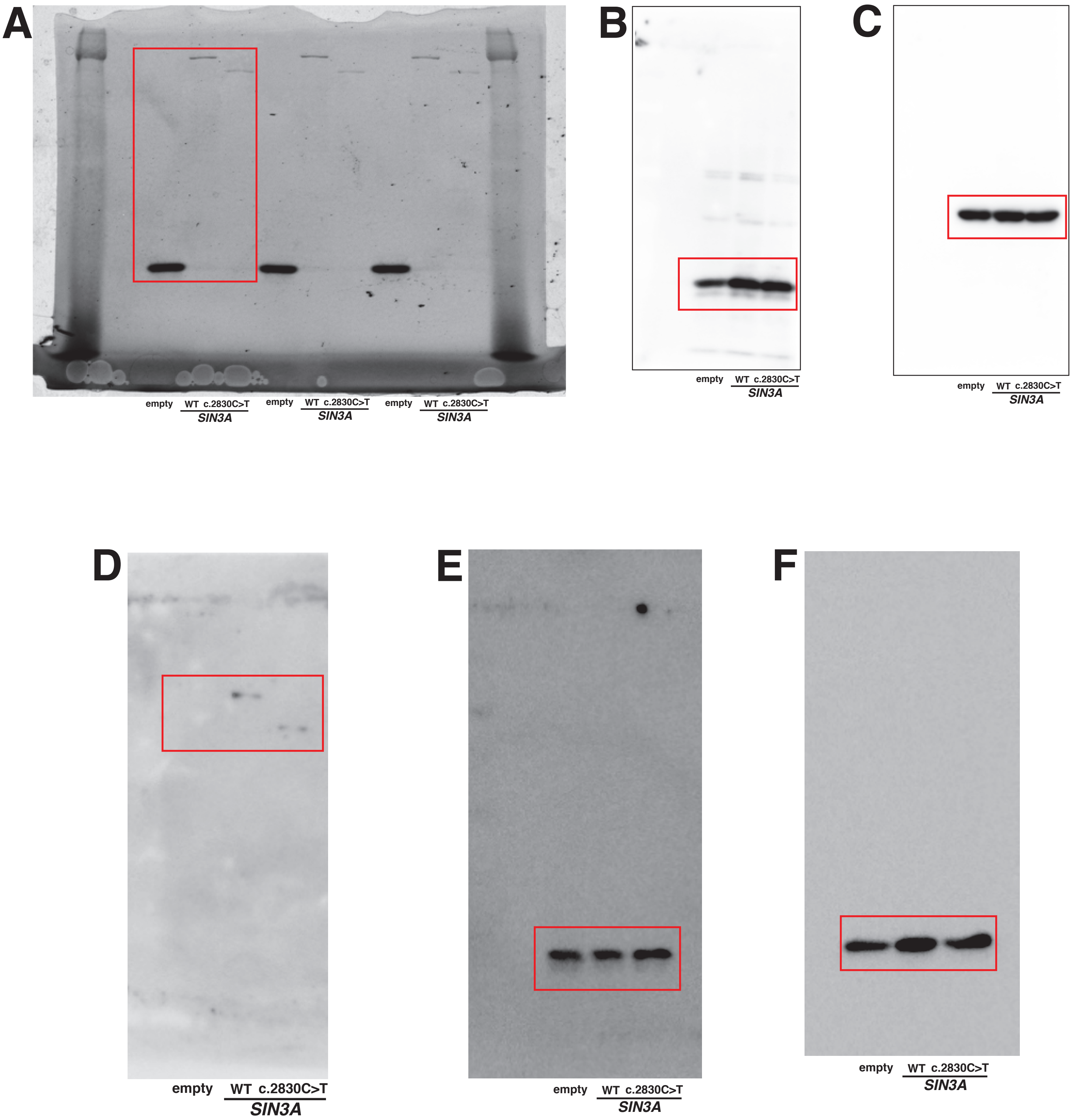

Supplementary Fig.8S

Expression of SIN3A p.Gln944* protein in MCF7 cells

(A-C) The full lengths of the gel and blots used in Figure 6 are shown, and the regions used in Figure 6 are indicated by red squares. The data were detected by Halo-tag ligand TMR (A), anti-GFP antibody (B) or anti-α-Actin antibody (C). (D-F) The full lengths of the blots used in Supplementary Fig.4S are shown, and the regions used in Supplementary Fig.4S are indicated by red squares. The data were detected by anti-SIN3A antibody (D), anti-GFP antibody (E) or anti-α-Actin antibody (F).
